# Rad50 zinc hook functions as a constitutive dimerization module interchangeable with SMC hinge

**DOI:** 10.1038/s41467-019-14025-0

**Published:** 2020-01-17

**Authors:** Hisashi Tatebe, Chew Theng Lim, Hiroki Konno, Kazuhiro Shiozaki, Akira Shinohara, Takayuki Uchihashi, Asako Furukohri

**Affiliations:** 1 Nara Institute of Science and Technology, Graduate School of Biological Sciences, Ikoma, Nara, 630-0192 Japan; 2 WPI Nano Life Science Institute (WPI-NanoLSI), Kanazawa University, Kakuma-machi, Kanazawa, 920-1192 Japan; 3 Department of Microbiology and Molecular Genetics, University of California, Davis, CA 95616 USA; 4 Institute for Protein Research, Osaka University, Suita, Osaka, 565-0871 Japan; 5 Department of Physics, Nagoya University, Nagoya, 464-8602 Japan; 6 Exploratory Research Center on Life and Living Systems (ExCELLS), National Institutes of Natural Sciences, 5-1 Higashiyama, Myodaiji, Okazaki, 444-8787 Japan

**Keywords:** Double-strand DNA breaks, Homologous recombination, Atomic force microscopy

## Abstract

The human Mre11/Rad50 complex is one of the key factors in genome maintenance pathways. Previous nanoscale imaging by atomic force microscopy (AFM) showed that the ring-like structure of the human Mre11/Rad50 complex transiently opens at the zinc hook of Rad50. However, imaging of the human Mre11/Rad50 complex by high-speed AFM shows that the Rad50 coiled-coil arms are consistently bridged by the dimerized hooks while the Mre11/Rad50 ring opens by disconnecting the head domains; resembling other SMC proteins such as cohesin or condensin. These architectural features are conserved in the yeast and bacterial Mre11/Rad50 complexes. Yeast strains harboring the chimeric Mre11/Rad50 complex containing the SMC hinge of bacterial condensin MukB instead of the RAD50 hook properly functions in DNA repair. We propose that the basic role of the Rad50 hook is similar to that of the SMC hinge, which serves as rather stable dimerization interface.

## Abstract (web summary)

The Mre11/Rad50 complex, which functions in genome surveillance, possesses antiparallel coiled-coil arms forming a ring-like structure similar to that of the SMC family proteins. Here the authors find that the Rad50 zinc hook functions similarly to the hinge of the SMC protein, and that the ring structure of the Mre11/Rad50 dimer also opens by disconnecting its globular head domains.

## Introduction

**Fig. 1:**
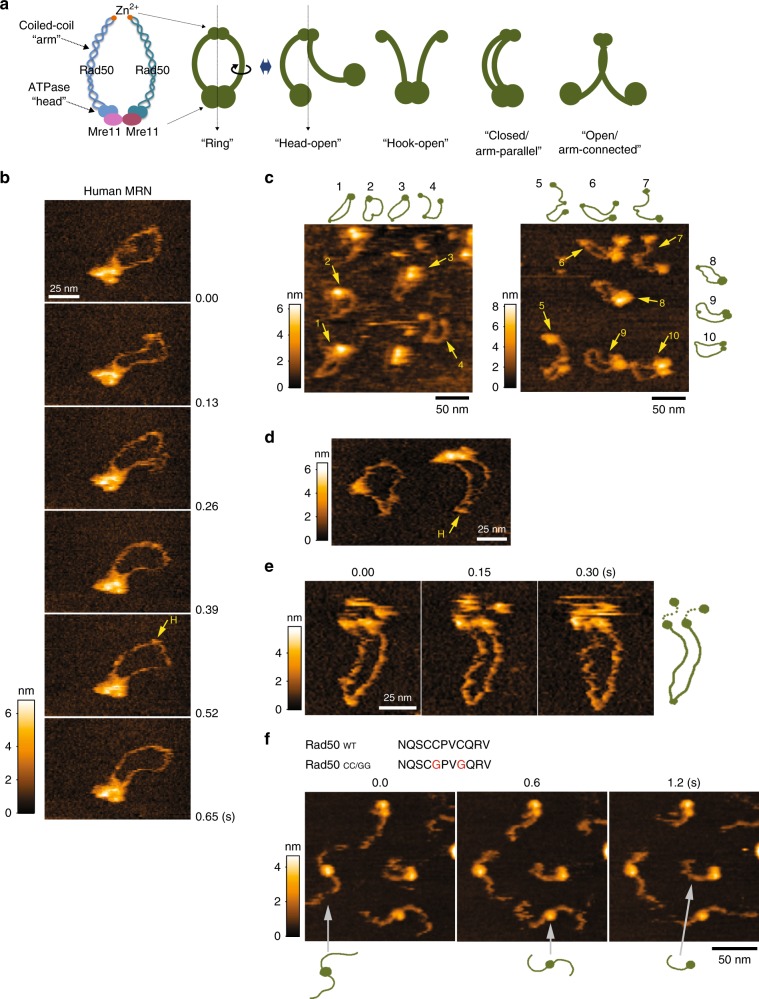
Dynamic structures of human Mre11/Rad50/Nbs1 complex in buffer captured by High-speed AFM. **a** A schematic representation of Mre11/Rad50 complex and its simplified version with five different structures (dark-green cartoons). A possible movement of the arm when the ring becomes the head-open is schematically depicted within the picture. **b** A flexible ring-shaped structure of human MRN in the presence of ATP-γ-S. Sequential images showing movement of coiled-coil arms. Imaging speed: 130 ms per frame. “H”: the hook. **c** Landscape images of MRN molecules on mica (−ATP). Presumed structures of MRN molecules are depicted around images. **d** MRN forming the ring (left) and the head-open (right) structure in the presence of ATP-γ-S. “H”: the hook. **e** Small globular molecules tethered to the head were visualized in the presence of ATP. The presumed structure of the middle image is depicted next to the images. Imaging speed: 150 ms per frame. **f** Amino acid sequence (681–691 a.a.) for the wild-type and mutant Rad50. In Rad50_CC/GG_, the two conserved cysteine residues were replaced with glycine (shown as red font). Sequential images show that two coiled-coil arms in the complex move independent of each other in the hook-deficient mutant Mre11/Rad50 (MR_CC/GG_) (−ATP). Imaging speed: 600 ms per frame.

The Mre11/Rad50 complex is known to have a characteristic architecture different from those of other nuclease complexes working in the genome maintenance pathways. Two molecules of the Mre11 exonuclease and two molecules of the ABC-type ATPase Rad50 form a unit of the Mre11/Rad50 (M_2_R_2_) complex (Fig. 1a)^1^. The overall structure of Rad50 is quite similar to that of the structural maintenance of chromosome (SMC) family of proteins such as cohesin and condensin. The N-terminal and C-terminal Walker A and B motifs of Rad50 form the “head” on which Mre11 is bound. With the Mre11 dimer, the two heads of Rad50 form a large globular domain in the M_2_R_2_ complex. The middle part of Rad50 forms the ~50 nm-long, rod- or string-like, anti-parallel coiled-coil structure called the “arm”. While the SMC proteins contain a “hinge” domain on the apex of the arm, Rad50 has the characteristic Zn^2+^-chelating CXXC motif called the “hook” on the apex of the arm; both the SMC hinge and the Rad50 hook function for the dimerization (see below). Therefore, there are two homo-dimerization sites locating both ends of the long coiled-coil arm of Rad50, which are believed to be important for the M_2_R_2_ complex to form a ring-shaped structure (Fig. 1a, “ring”).

The Mre11/Rad50 complex is found in all the three domains of life from bacteria and archaea to human, although the eukaryotic complex is known to have the third subunit Nbs1/Xrs2, which is important for DNA damage checkpoint activation^1,2^. Mre11 as well as Rad50 from various organisms have similar biochemical characteristics, and the structure of each domain of the two proteins is also well conserved among species^1,3–7^. On the other hand, previous studies to visualize the entire architecture of the complex by atomic force microscopy (AFM) showed that the shapes of the eukaryotic complex are quite different from those of the archaeal and bacterial complexes^8,9^. One of the major shapes for the human and yeast complexes was “hook-open” (M_2_R_2_ connected only via the head, see Fig. 1a), but such a hook-open conformation was not observed with the archaeal and bacterial complex^9^, though the Rad50 hook is highly conserved through evolution^10,11^. Other groups also reported that the hook-open structure of human Mre11/Rad50 was evident by conventional AFM observation in air^12,13^. In addition, human Mre11/Rad50 transiently opening and closing the coiled coil arms at the hook was visualized by AFM observation in solution^14^. The reported hook-open structures are puzzling because the genetic analyses showed that the hook is essential for the Rad50 function in vivo^10,15–18^. To explain this discrepancy, it has been proposed that the opened hooks of two M_2_R_2_ complexes bound to different DNA ends can assemble to form the (M_2_R_2_)_2_ complex that tethers DNA strands^10,19^. Although accumulating data point to the biological significance of the Mre11/Rad50 complex in tethering and stabilizing DNA, it is still unclear if the Mre11/Rad50 complex carries out the tasks by forming the inter-molecular complex mediated by the hook opening/closing.

Recent advances in observation of biomolecules using high-speed atomic force microscopy (HS-AFM) have started revealing the shapes and dynamic motions of flexible proteins at nanometer spatial and sub-second time resolutions^20^. We employed this novel technique to directly visualize the human Mre11/Rad50/Nbs1 (MRN) complex as well as the yeast and bacterial Mre11/Rad50 complexes in solution. Despite what has been reported in the previous observations^8,9,12–14^, we found that the Mre11/Rad50 ring is consistently closed at the hook, while occasionally opens at the head, like in other SMC proteins. Observed similarities between the structures of Mre11/Rad50 proteins and those reported for the SMC proteins suggest that, like the SMC hinge, the Rad50 hook mainly works as a dimerization module. This model is further corroborated by in vivo experiments showing that the chimeric Rad50 protein harboring the SMC hinge of bacterial condensin MukB, instead of the hook, fully functions in repairing DNA damages in yeast.

## Results

### Direct visualization of human Mre11/Rad50/Nbs1 by HS-AFM

To observe the human MRN complex under a physiologically relevant condition, we directly visualized it on bare mica surface in a buffer by using HS-AFM. The characteristic ring-shaped structures reported previously^8,9,14^ (hereafter called “ring”) were observed (Fig. 1a, b). Two ~50-nm-long arms protruded from the large globular structure, the supposed head domains that consist of two Rad50 ATPases bound to the Mre11 dimer. The two arms moved flexibly in solution while connected at one end, representing that the Rad50 coiled-coil arms are bridged by the zinc hooks (Fig. 1b, Supplementary Movie 1). In addition to the ring, the “head-open” structure was frequently observed (Fig. 1c) and these two major structures were observed either in the presence or absence of ATP/ATP-γ-S. In the head-open structure, two Mre11/Rad50 heterodimers were linked only at the hook and the two globular domains were separated. Judging from the curve of each arm, one of the two arms seems to be turned over in some of the head-open molecules (Fig. 1a, c, d, ring to head-open). Other structures, such as multimers and monomers, were observed less frequently than the dimeric form (e.g., Supplementary Movie 2, Supplementary Fig. 1c).

Because the expression of the Nbs1 protein was low and the complex formation of Mre11/Rad50 with Nbs1 was not efficient, in two independent preparations only a portion of our MRN contained the Nbs1 subunit (Supplementary Fig. 1a). We occasionally observed one or two small globular structures tethered to the globular domain of the complex via a thin flexible linker (Fig. 1e, Supplementary Movie 3, Supplementary Fig. 1d). To our knowledge, there is no report of direct visualization of Nbs1, but the C-terminal half of Nbs1 is predicted to have a highly flexible region that tethers Nbs1 to the Mre11/Rad50 complex;^21^ one possible interpretation is that the small globular structures correspond to the Nbs1 bound to the Rad50 head through the flexible C-terminus linker. However, visualization of these hanging small structures was quite difficult, as most of them moved around faster than the imaging speed, hampering further analyses of the precise shape and origin of this structure.

It is surprising that major structures of the human MRN complex are the ring and the head-open, because the major shapes of the human MRN complex previously reported were the ring and the hook-open structures, and in the latter case, the M_1_R_1_ subcomplex is dimerized only via its globular domain (Fig. 1a, hook-open)^8,9,13,14^. We tried to detect the hook-open structure but hardly observed it (e.g., 0/25 molecules in Supplementary Movie 2, 2/221 molecules in Supplementary Fig. 1c, MRN [−ATP]). The possibility that the globular domain was misinterpreted as the hook was ruled out by observing the mutant Mre11/Rad50 complex with glycine substitution of the Cys residues in the conserved CXXC motif in the Rad50 hook (Fig. 1f). As expected, the hook-open structure was dominantly observed with the mutant complex whereas the ring structure was not. The two coiled-coil arms of the Mre11/Rad50_CC/GG_ complex protruded from the globular domain in opposite directions and moved independently (Supplementary Movie 4), suggesting that the globular domain tends to spread the arms. This kind of wide-open arms and their independent movements were not seen with the head-open structure of the wild-type Mre11/Rad50 complex.

### Human Mre11/Rad50 flexibly changes its structure in solution

**Fig. 2:**
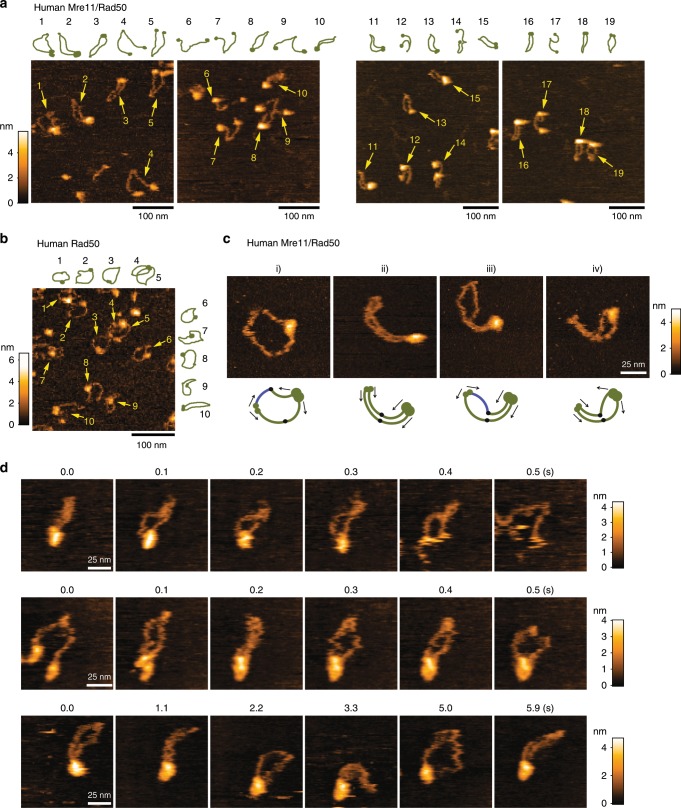
Structural variations of human Mre11/Rad50 and their exchanges video-imaged by high-speed AFM. **a** Landscape images containing the ring and head-open structures of human Mre11/Rad50 (left: −ATP; right: +ATP). Presumed structures of Mre11/Rad50 molecules are depicted with a dark-green line around images. **b** A landscape image of human Rad50 (−ATP). **c** Representative ring structures observed for human Mre11/Rad50 (−ATP). A schematic diagram of presumed structures is shown below. In (i) and (iii), one of the arms (blue line) seemed to be turned over and twisted 180 degrees at a particular position (black oval). **d** Movement of human Mre11/Rad50 was real-time imaged by directly adding proteins into the imaging chamber while imaging at 100 ms per frame (+ATP). Scan size, 100 × 100 nm. Upper: sequential images of Mre11/Rad50 opening the ring. Middle: the same Mre11/Rad50 closing the ring. Lower: selected images from the movie showing the ring and the closed/arm-parallel structures.

To test whether Nbs1 affects the structure of the Mre11/Rad50 complex, the human Mre11/Rad50 complex without Nbs1 was observed. As seen with the MRN complex, both ring and head-open structures were observed for the complex lacking Nbs1 (Fig. 2a). Previous structural analyses showed that the Mre11 homodimer tethers the Rad50 head domains^3,4^. Therefore, we asked if the head-open structure is formed when the Mre11 subunit is lost from the complex. The molar ratio of Rad50 to Mre11 in purified MRN and Mre11/Rad50 preparations was approximately 1:1 (Supplementary Fig. 1b), suggesting that the complex we prepared contained about the same number of Rad50 and Mre11 molecules. In addition, the majority of purified Rad50 took the ring-shaped structure (Fig. 2b), demonstrating that the Rad50 head itself can dimerize even in the absence of Mre11 (Supplementary Fig. 1c).

Figures 1 and 2a–c show what happened when we first incubated the human MRN or Mre11/Rad50 complex on mica and observed the complex partially bound on the mica surface. Dynamic conformational changes of the ring structures were observed as shown in Fig. 2c and Supplementary Movie 5 and S6. In Fig. 2c, the ring appeared to be a circle (Fig.  2c-i) but occasionally the hook was entirely folded and the two arms were parallel (Fig.  2c-ii). In addition, the globular domain also seems to change its structure and accordingly the arms either were spread and protruded from the head at large angles (Fig.  2c-i, iv) or were parallel near the head (Fig.  2c-i, iii). In Fig. 2c-i, one arm appears to be bent like an elbow at a particular position in the middle (shown as a black oval in the model), probably because there is a relatively flexible region at the position and a weak adsorption to mica may make one arm kinked or twisted. Consistent with our observation, coiled-coil free regions are predicted to increase a local flexibility in the arm^12^. The presence of the elbow-like structure was also reported for the SMC family proteins and this morphological characteristic is implicated in their actions on DNA^22^.

To eliminate the possibility that our experiments observed structures deformed by strong attachment of the proteins to the mica surface, we next carried out a video-imaging of human Mre11/Rad50 by adding the protein into the imaging chamber while scanning the mica surface to capture the movement of the protein complex before it completely attaches to mica. While the Mre11/Rad50 was tumbling on mica, its arms flexibly moved by Brownian motion and the protein ring reversibly opened and closed at the head domain (Fig. 2d). The entire structure also changed dynamically and the ring, head-open, and “closed/arm-parallel” structures were frequently observed.

Although the Mre11/Rad50 complex changed its shape dynamically, once the ring was formed it tended to persist for a while, suggesting that the globular structure composed of Rad50 heads and a Mre11 dimer is stable to some extent. The ring form seems to be preferable to the head-open form for MRN on mica (Fig. 2a, Supplementary Fig. 1c). Crystal structure and SAXS studies showed that ATP binding closes the Rad50 head dimer^4,6,23,24^. When the MRN or Mre11/Rad50 was pre-incubated with ATP, the head-open molecules were still detected together with the ring (Supplementary Fig. 1c).

The Mre11/Rad50 complex has previously been reported to flexibly change its structure^8,12^. Our observation more clearly visualized how plastic and flexible the entire protein ring of Mre11/Rad50 is in the solution. To quantify the flexibility of the global coiled-coil-arm region of MRN, we estimated persistence lengths for the ring and the head-open forms on mica. The persistence lengths calculated with the two-dimensional worm-like-chain (WLC) model Eq. (1) were ~22 nm for the ring and ~25 nm for the head-open (Supplementary Fig. 1e), which are values similar to those previously evaluated using AFM in air (30 nm)^12^. Similar persistence lengths for two conformations suggests that the dimerization of the head has little effect on the flexibility of the coil–coil arms.

Although the hook-open structure has been believed to be the major form of the Mre11/Rad50 complex^8,9,12–14^, such a structure was rarely observed in our experiments even when we used the buffer conditions similar to those used previously^14^. Two possible reasons can be considered: the procedure fixing the complex to a mica substrate, and the fixation strength. Some previous AFM works in air required washing samples with water after the molecules were adsorbed on a mica to avoid segregation of salts contained in the protein solution^8,9,12,13^ but the exposure of the complex to water could lead a protein denaturation (Supplementary Fig. 2a). Although some observations were carried out in a buffer^12,14^, the imaging speed was much slower (39 s per frame) than ours (~100 ms per frame), making it impossible to observe molecules weakly adsorbed onto the mica substrate and thus mobile. When we strongly fixed the MRN complex on a mica surface treated with an (3-Aminopropyl)triethoxysilane (APTES-mica) or crosslinked the complex onto the APTES-mica by glutaraldehyde, pairs of coiled-coil arms were attached to each other and two or more complexes often aggregated via their head domains (Supplementary Fig. 2a). Those strongly fixed complexes look like the complex in the hook-open form. On the other hand, the HS-AFM used in this study allows observation of proteins that only weakly or partially attach to mica, enabling us to observe native structures of Mre11/Rad50 complex. Notably, because the Mre11/Rad50 complex in the solution keeps moving so quickly and flexibly, we sometimes obtained images showing disconnection of the ring even under the imaging as seen in Supplementary movie 5; one frame showed disconnected coiled-coil arms and the next frame showed the two arms similarly curved but they formed a ring-like shape, showing that actually the ring is intact within the observation time. If the coiled-coil/hook/head is in fact disconnected, each coiled-coil arm should immediately spread and start moving independently of each other like those in mutant Mre11/Rad50_CC/GG_ did (Supplementary Movie 4).

### The conformational features of Mre11/Rad50 are conserved

**Fig. 3:**
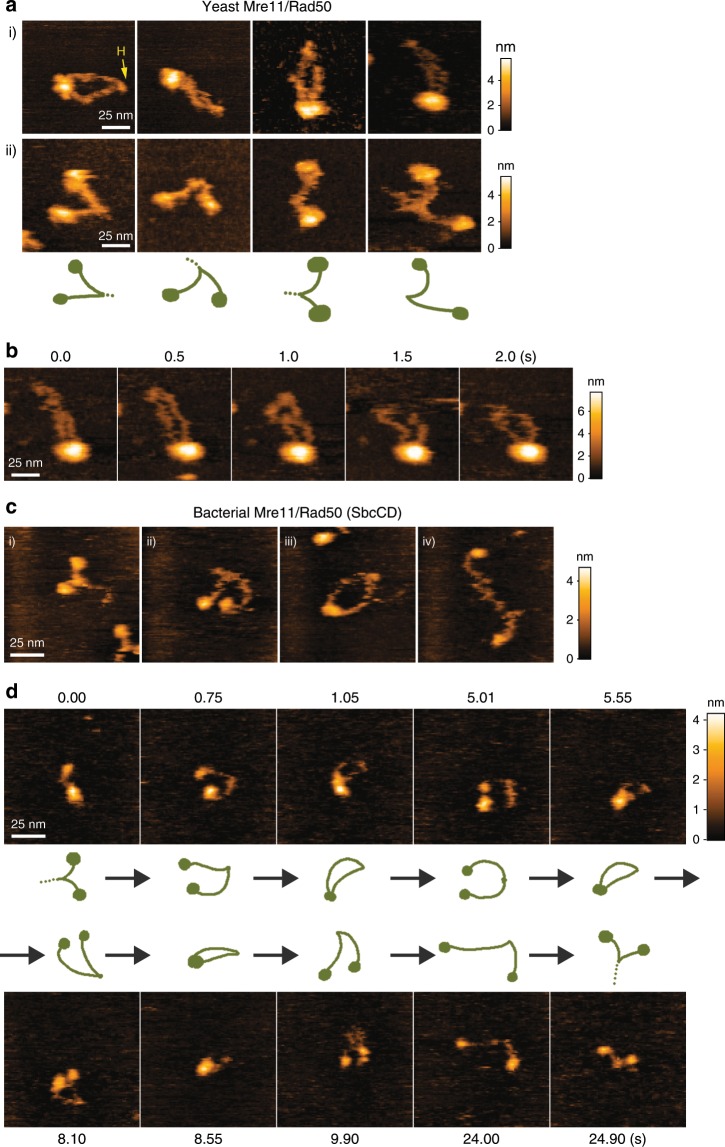
*S. pombe* Mre11/Rad50 and *E. coli* Mre11/Rad50 (SbcCD) are structurally similar to human Mre11/Rad50. **a** (i) ring and (ii) open/arm-connected structures of *S. pombe* Mre11/Rad50 (−ATP). “H”: the hook. **b** Sequential images showing movement of coiled-coil arms of *S. pombe* Mre11/Rad50. Imaging speed: 500 msec per frame. **c** Various structures observed with *E. coli* SbcCD (−ATP). (i) open/arm-connected, (ii) head-open, (iii) ring, and (iv) “S-shaped” structures. **d** SbcCD was real-time imaged by adding the protein to the imaging chamber while scanning (−ATP). The structural transients are shown. Imaging speed: 150 msec per frame. Scan size, 100 × 100 nm. Representative images selected from the movie of a single SbcCD molecule (Supplementary Movie 7) are shown with schematic diagrams of presumed structures.

Next we analyzed the fission yeast (*S. pombe*) Mre11/Rad50 under the same conditions used for the human Mre11/Rad50. The overall architecture of the yeast Mre11/Rad50 is remarkably similar to that of the human Mre11/Rad50 (Fig. 3a-i, ring, Fig. 3a-ii, head-open, 3b, ring). It seems that the arms of the head-open structure are shorter than those of the ring structure (compare Fig. 3a-i, ii), implying that the two arms are partially bound to each other (Fig. 1a, “open/arm-connected”) and were invisible probably because they did not attach to mica. Importantly, like human Mre11/Rad50, the yeast complex did not exhibit the hook-open structure as a major form under the experimental conditions tested.

In a previous report^9^, the ring was observed with Mre11/Rad50 from humans, yeast, and archaea, but not bacteria. Therefore, we further analyzed the structure of the bacterial Mre11/Rad50 complex by HS-AFM. Purified *E. coli* Mre11/Rad50 (SbcCD) showed a characteristic structure in which two coiled-coil arms are engaged toward their middle and the rest of the arms sharply curved to widely separate the two globular domains (Fig. 3c-i), resembling the open/arm-connected structures of yeast Mre11/Rad50. The entire arm length was also difficult to be detected, probably because the two arms were bonded to each other and did not attach to mica. The arms were shorter, curved and seemed to be less flexible than those of the human and yeast Mre11/Rad50. Most of the SbcCD molecules were observed as this open/arm-connected structure in the presence or absence of ATP. We also observed arm-detached structures that remarkably resemble the human head-open Mre11/Rad50 (Fig. 3c-ii). They transiently turned to the ring as the eukaryotic complexes do (Fig. 3c-iii), although the ring was detected much less frequently than with the eukaryotic complexes. Conversions between the head-open and ring structures were repeatedly observed (Fig. 3d, Supplementary Movie 7). When we incubated SbcCD on mica prior to the observation, we observed the S-shaped SbcCD with its arms tightly bound to the mica surface (Fig. 3c-iv). Thus, the hook region of SbcC (*E. coli* Rad50) is likely to be flexible enough to twist, like the human complex does.

### Rad50’s zinc-hook is interchangeable with the SMC hinge

**Fig. 4:**
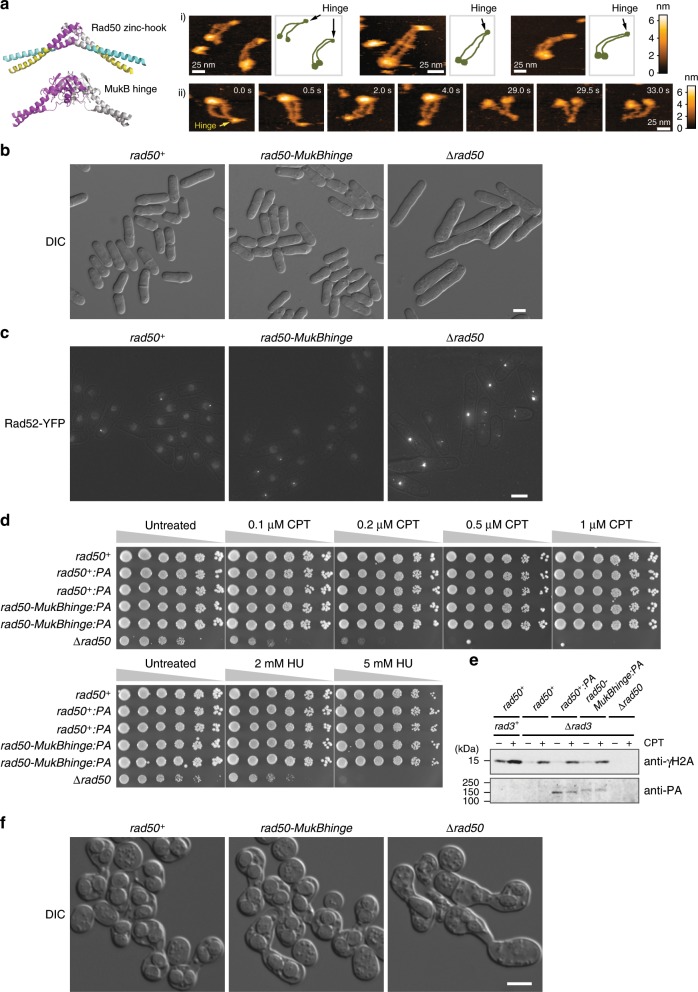
Chimeric Mre11/Rad50 harboring MukB SMC hinge instead of Rad50 zinc hook efficiently functions in repairing DNA damages in vivo. **a** Left: structural comparison of the *P. furiosus* Rad50 zinc-hook (upper; PDB 1L8D) and the *E. coli* MukB hinge (lower; PDB 2WMM). The regions shown in magenta and gray were replaced between the *S. pombe* Rad50 zinc-hook and the *E. coli* MukB hinge. The N-terminal and C-terminal coiled-coil regions in the zinc-hook structure are colored in cyan and yellow, respectively. Right: (i) the ring and the head-open structures of *S. pombe* Mre11/Rad50-MukBhinge (−ATP). (ii) Representative images showing Mre11/Rad50-MukBhinge opening the head. Imaging speed: 500 ms per frame. **b** Fission yeast *rad50-MukBhinge* cells do not exhibit cell elongation phenotypes. A *rad50*^*+*^ strain (*rad50*^*+*^*:PA*, HT1697), a *rad50-MukBhinge* strain (*rad50-MukBhinge:PA*, HT1683), and a *rad50* deletion strain (*∆rad50*, HT1250) were grown in YES, followed by observation with DIC microscopy. The scale bar: 5 µm. **c** Rad52 foci do not increase in *rad50-MukBhinge* cells. Formation of Rad52 foci was monitored in the following strains carrying the *rad52*^*+*^*:YFP* allele: *rad50*^*+*^ (*rad50*^*+*^*:PA*, HT1792), *rad50-MukBhinge* (*rad50-MukBhinge:PA*, HT1791), and ∆*rad50* (*∆rad50*, HT1790). Rad52-YFP projection images that are superimposed on bright field microscopy images are presented. The scale bar: 5 µm. **d** Fission yeast *rad50-MukBhinge* strains grow as well as wild-type strains in the presence of genotoxins. Sensitivities to CPT and HU were examined in the following strains: an untagged *rad50*^*+*^ wild-type strain (KS1598), two PA-epitope tagged *rad50*^*+*^ strains (HT1633, HT1697), two PA-epitope tagged *rad50-MukBhinge* strains (HT1655, HT1683), and a *∆rad50* deletion strain (HT1250). **e** γH2A levels are normal in fission yeast cells carrying the *rad50-MukBhinge*. γH2A levels were examined by immunoblotting using strains of the following genotypes: *rad3*^*+*^ *rad50*^*+*^ (KS1598), *∆rad3 rad50*^*+*^ (FY32725), *∆rad3 rad50*^*+*^*:PA* (HT1770), *∆rad3 rad50-MukBhinge:PA* (HT1768), and *∆rad3 ∆rad50* (HT1782). Source data are provided as a source data file. **f** *rad50-MukBhinge* homozygotes sporulate with no visible defect. Mating, meiosis, and sporulation were induced between the following pairs of strains of the opposite mating types: *rad50*^*+*^*:PA* (HT1693, HT1697), *rad50-MukBhinge:PA* (HT1680, HT1683), and *∆rad50* (HT1250, HT1257). Sporulation was observed in DIC microscopy. The scale bar: 5 µm.

Our HS-AFM observation showed that the Rad50 dimer is persistently linked at the hook, while the ring occasionally opens at the head. Interestingly, this structural feature is similar to that of the SMC proteins. The observed structural similarity led us to hypothesize that the zinc-hook can be replaced by the SMC hinge. A previous attempt to substitute the zinc-hook of budding yeast Rad50 with the *Thermotoga maritima* SMC hinge resulted in little success, possibly because the SMC hinge from the thermophilic bacterium might function inefficiently at 30 °C used for the experiment in yeast^16^. Therefore, we took another approach where the zinc-hook of fission yeast Rad50 was replaced with the hinge domain of *Escherichia coli* MukB, the bacterial condensin SMC subunit that homo-dimerizes via its hinge (Fig. 4a, left, Supplementary Fig. 2b, hereafter the chimeric Rad50 is called Rad50-MukBhinge)^25^.

When the fission yeast Mre11/Rad50-MukBhinge complex was purified and observed by HS-AFM, it exhibited ring and head-open structures similar to those of the Mre11/Rad50 complex (Fig. 4a-i, ii). The Mre11/Rad50-MukBhinge complex also showed an additional small globule at the apex of the coiled-coil arms, where the zinc-hook was replaced with the SMC hinge. A very similar structure was reported for the hinge domain of the eukaryotic condensin SMC dimer^26^. Therefore, we concluded that the yeast Rad50-MukBhinge protein successfully dimerized to connect the two coiled-coil arms via the substituted MukB hinge.

To further test the functionality of the Rad50-MukBhinge chimera, a fission yeast strain whose chromosomal *rad50*^*+*^ gene was replaced with the *rad50-MukBhinge* allele was constructed. The *rad50-MukBhinge* cells were morphologically indistinguishable from wild-type *rad50*^*+*^ cells, in contrast to the highly elongated *rad50* null (*∆rad50*) mutant cells (Fig. 4b)^27^. Whereas increased Rad52 foci formation, an indicative of DNA repair defects^28^, was apparent in ∆*rad50* mutant cells (Rad52 foci positive cells: 64.2% (138/215) for ∆*rad50*; 12.2% (30/246) for *rad50*^*+*^), *rad50-MukBhinge* cells did not exhibit such an increase like *rad50*^*+*^ strain (12.7% (31/245)) (Fig. 4c). ∆*rad50* cells exhibited apparent growth defects without DNA damaging agents and showed severe sensitivities to clastogens inducing DNA double-strand breaks, such as camptothecin (CPT) and hydroxyurea (HU) (Fig. 4d)^24,29,30^. Indeed, unlike the *∆rad50* mutant, the *rad50-MukBhinge* strain grew normally in untreated conditions and was resistant to both CPT and HU (Fig. 4d), indicating that DNA breaks are efficiently repaired in the *rad50-MukBhinge* strain. We found that chimeric Rad50 protein harboring *Bacillus subtilis* SMC hinge also functions in repairing DNA damage in the *rad50-BsSMChinge* strain (Supplementary Fig. 3a, b). On the other hand, the Rad50_CC/GG_ protein, whose zinc-hook is impaired (Fig. 1f)^31^ was found to be defective and failed to complement the *∆rad50* phenotypes, confirming that dimerization via the hook is essential for Rad50 function^10,18,29^ (Supplementary Fig. 3a, b).

In addition to its involvement in homology-directed DNA repair, the Mre11/Rad50 complex plays a crucial role in the repair of double-strand breaks through activation of the DNA damage checkpoint kinase Tel1/ATM (ataxia telangiectasia mutated) in fission yeast^1,32^. To further examine whether the Rad50-MukBhinge chimera retains the ability to mediate the Tel1-dependent phosphorylation of histone H2A^33^, the level of phosphorylated H2A (γH2A) was monitored in the absence of Rad3/ATR, another checkpoint kinase with a redundant role in H2A phosphorylation^33^. Without DNA damaging reagents, γH2A was barely detectable in both the *rad50*^*+*^ and *rad50-MukBhinge* strains (Fig. 4e). Upon exposure to CPT, γH2A levels were elevated comparably in *rad50*^*+*^ and *rad50-MukBhinge*, indicating that Rad50-MukBhinge activates the Tel1/ATM kinase as efficiently as wild-type Rad50 does (Fig. 4e, Supplementary Fig. 2c). As reported previously^29^, γH2A was completely abolished in the *∆rad3 ∆rad50* double deletion mutant.

Rad50 is essential for meiotic recombination, and hence gametogenesis, in a wide variety of eukaryotes, including fission yeast^34,35^. We found that *rad50-MukBhinge* cells were fertile, producing healthy, viable spores (Fig. 4f, Supplementary Fig. 3c). Collectively, all the analyses of the Rad50-MukBhinge chimera indicated that the Rad50 zinc-hook is structurally as well as physiologically interchangeable with the SMC hinge. The assumed function of the SMC hinge, that is to stably link two coiled-coil arms, is also likely to be an essential function of the Rad50 hook in repairing double-strand breaks in vivo.

## Discussion

In this paper we visualized the human, yeast, and bacterial Mre11/Rad50 complexes by HS-AFM. They dynamically change their shapes, and along with flexible movements of coiled-coil arms, the zinc hook and surrounding regions also change their structures. An unexpected finding is that the human Mre11/Rad50 ring repeats open-close actions at the head, while the hook is persistently closed. The yeast and bacterial Mre11/Rad50 complexes also open their ring only at the head domains. Consistent with our observations, reported electron microscopy images of *S. cerevisiae* and *E. coli* Mre11/Rad50 include similar head-open structures^36,37^. On the other hand, previous studies provided images of the human Mre11/Rad50 ring opened at the hook even in the absence of DNA^8,9,12–14^. It was also reported that, when Mre11/Rad50 binds to DNA with its head domains, the coiled-coil arms become parallel and bridge the M_2_R_2_ complexes bound to different DNA strands^14^. These studies led to the model that DNA binding induces the conformational change in Rad50 head domains, altering the orientation of the coiled-coil arms to open the hook, which is then used for the intermolecular assembly of two M_2_R_2_ complexes^14,19^. In contrast, we found that the intermolecular interactions of M_2_R_2_, including multimerization, were mostly between the globular domains (Supplementary Fig. 2d).

Currently, we cannot completely exclude the possibility that the hook opens when Mre11/Rad50 binds to DNA; however, it seems unlikely because our genetic data show that the SMC hinge can replace the hook in Rad50. This observation is surprising, as the Rad50 hook and the SMC hinge are structurally quite different. The Rad50 hook is composed of a set of cysteine residues within a short hairpin loop (14 amino acids, Fig. 4a, left). The Zn^2+^-mediated dimerization via the hook is extremely stable because the hook peptide has a very high affinity for Zn^2+^ in the femtomolar range, much lower than the cellular Zn^2+^ concentration that is within the picomolar range^38,39^. On the other hand, the SMC hinge is composed of 150–200 amino acids residues and the two hinge domains generate the stable doughnut-like dimer that has an affinity to DNA (Fig. 4a)^40^. Indeed, controlled resolution of such different dimerization modules during the in vivo actions of Rad50 is very difficult to imagine.

A possibility that both the hook and the hinge opens cannot be completely ruled out at present. If the hook-opening in Rad50 is necessary for Mre11/Rad50 to function, our result would indicate that the SMC hinge also opens, as opposed to what has been believed; to our knowledge, there is no direct visualization by AFM or electron microscopy that the SMC hinge actually opens. However, there are contradictory reports about possible opening of the SMC hinge. Cohesin still functions even if Smc3 is fused to Scc1 (or Scc1 to Smc1), suggesting that cohesin may transiently open at the SMC hinge if the ring must open to function in vivo^41^. However, others have reported that the main DNA entry gate is the head^42^. Another possibility is that both the Mre11/Rad50 and SMC proteins function without opening their rings. Interestingly, *B. subtilis* can grow normally even if its SMC hinge was exchanged with the *Pyrococcus furiosus* Rad50 hook if the coiled-coil arms are of a proper length^43^. Given these results and our current findings, the Rad50 hook and the SMC hinge can be considered as similar architectural dimerization module rather than the specific function unit. Further comparison of the structures of Mre11/Rad50 and SMC proteins will provide a novel insight into how these characteristic proteins function to tether DNA strands in vivo.

## Methods

### Fission yeast strains and general techniques

Media, reagents, and basic techniques for yeast genetics, such as transformation and genome editing, have been described elsewhere^44^. Fission yeast strains used in this study are listed in Supplementary Table 1. Unless explicitly indicated, fission yeast cells were grown at 30 °C in the YES complete medium or in the EMM minimal medium. Sensitivity to genotoxins was analyzed by spotting 5-fold serial dilutions of exponentially growing cells onto YES or EMM agar plates containing the indicated amounts of camptothecin (CPT) or hydroxyurea (HU). Photos were taken after 4-day incubation at 30 °C.

Mating, meiosis, and sporulation were induced by 2–4 days incubation at 25 °C on malt extract agar or synthetic sporulation agar^45^. Before plating spores, cells and asci were lysed by overnight incubation in 1% glusulase (PerkinElmer, NEE154001EA); remaining spores were counted in hemocytometer; twenty thousand spores were spread per YES plate. After 24 h incubation at 30 °C, spores were observed microscopically to determine spore viability; spores were judged viable when they formed microcolonies containing four or more cells^46^. Approximately 400 spores were examined in each analysis.

Activation of Tel1 kinase was induced by the addition of CPT. Fission yeast cells were grown at 30 °C in the YES liquid medium to early-mid log phase (2.0–4.0E+6 cells per ml), followed by 1-h incubation in the presence of 2.5 µM CPT. For crude cell extract preparation, harvested cells were broken with glass beads in the presence of 10% TCA, followed by protein extraction with 1× SDS-PAGE sample buffer. For detecting γH2A, total protein in the crude cell extract was separated by SDS-15%PAGE and analyzed by Western blotting using anti-phospho H2A (S129) antibodies (Abcam, ab17353) and goat anti-rabbit IgG IRDye 800CW conjugated (Abcam, ab216773). Signals at 800 nm were detected and quantified with an Odyssey imaging system (Li-COR). The same membrane was used for detecting PA-epitope tagged wild-type Rad50 or Rad50-MukBhinge using rat anti-PA tag antibody (Wako, 012-25863), donkey anti-rat IgG antibody HRP (Jackson, 712-035-150), ECL prime (GE healthcare) and ImageQuant LAS4000 (GE Healthcare). Images were further processed for figure preparation with Adobe Photoshop Elements. The uncropped images are provided in a source data file.

### Microscopy

To observe exponentially growing fission yeast cells in differential interference contrast (DIC) microscopy, cells were placed on thin YES agarose film under the cover slip. To examine asci and spores in DIC microscopy, they were resuspended in water under the cover slip. DIC microscopy was performed at room temperature (approximately 23 °C) with a Nikon Eclipse E600 microscope and a PlanApo ×60 (NA 1.4) objective lens. DIC images were taken using a digital CCD camera ORCA-RE C4742-80-12G (Hamamatsu Photonics) under the control of Openlab software (Improvision). To monitor foci formation of Rad52-YFP in fluorescence microscopy, fission yeast cells that were grown in the EMM liquid medium to early-mid log phase (approximately 2.0E+6 cells per ml) were collected by centrifugation and placed on a thin EMM agarose film under the cover slip. Fission yeast cells growing on EMM agar at room temperature (approximately 23 °C) were observed with fluorescence microscopy. Fluorescence microscopy was performed using the DeltaVision Elite system equipped with a PlanApo ×60 (NA 1.42) objective lens (GE Healthcare). Rad52-YFP images were taken at 0.4-µm steps along the *z*-axis. Images were deconvolved with softWoRx software (GE Healthcare), after which *z*-axial projection images were produced with the “Projection type” set “Max intensity” in softWoRx or FIJI^47,48^. Images were further processed for figure preparation with Adobe Photoshop CS6.

### Protein purification from Sf9 cells

Human MRE11 cDNA was cloned into pFASTBAC1. Human NBS1 cDNA was cloned into pFASTBAC HTA or pFASTBAC1 to produce N-terminal histidine tagged or no tagged protein. Human RAD50 cDNA fused with C-terminal Prescission protease cleavage site and 3×FLAG tag sequences was also cloned into pFASTBAC1. Following manufacturer’s instructions, recombinant bacmid DNA containing human Mre11, His_6_-Nbs1, or Nbs1 and Rad50-3×FLAG expression cassettes was produced and transfected into Sf9 cells to obtain each recombinant baculovirus. For expression, Sf9 cells were infected with these baculoviruses and incubated for at 28 °C for 50 h. Cells expressing proteins were resuspended in Lysis buffer H (50 mM potassium phosphate pH 7.0, 20 mM 2-mercaptoethanol, 10% glycerol, 0.5% Tween 20) supplemented with 0.5 M KCl and Protein Inhibitor Cocktail (Wako, #160-26071). After sonication treatment, cell extracts were prepared by centrifugation and diluted by the above buffer to make the final concentration of 200 mM KCl. Cell extracts were subjected onto anti-FLAG M2 beads (Sigma, #A2220) and bound proteins were eluted by PreScission Protease cleavage (GE Healthcare, #27084301). The fraction containing Mre11/Rad50 or MRN was loaded onto HiTrapQ in Buffer A (25 mM Tris-HCl pH 8.0, 100 mM NaCl, 1 mM DTT, 10% glycerol) and proteins were eluted via a linear gradient (100 mM–1 M NaCl). Wild-type Mre11/Rad50 was further purified by using a Superdex 200 column equilibrated with Buffer B (25 mM Tris-HCl pH 8.0, 500 mM NaCl, 1 mM DTT, 10% glycerol. Peak fractions were pooled, aliquoted, frozen by liquid nitrogen and stored at −80 °C. MRN_notag_ was used the experiments producing the data for Supplementary Fig. 1c–e, 2a, and MRN_His_ was used for the other experiments.

### Protein purification from yeast cells

A wild-type strain (CA3) was co-transformed with the pREP1-rad50:TEV:PA and pREP1-mre11:HRV3C:3xFLAG and grown at 30 °C in EMM without thiamine to induce exogenous *S. pombe* Mre11/Rad50 expression. Cells were then collected, frozen and lysed in Lysis buffer Y (20 mM potassium phosphate pH 7.4, 300 mM KCl, 10% glycerol, 1 mM DTT, 0.25% Tween 20) supplemented with Protein Inhibitor Cocktail (Wako, #160-26103) and the final concentration of 1 mM PMSF using Multi-Beads Shocker (Yasui Kikai). Cell lysate was subjected to anti-FLAG affinity purification using anti-FLAG M2 agarose beads (Sigma, A2220). After the removal of 3xFLAG tag by HRV3C protease, eluted proteins were collected. Wild-type proteins were further purified by size exclusion chromatography using Superose 6HR 10/30 (GE healthcare) in Buffer C (20 mM potassium phosphate pH 7.4, 200 mM KCl, 10% glycerol, 0.01% NP-40, 1 mM DTT).

### Preparation of *E. coli* SbcCD

SbcCD proteins used in this study were purified and analyzed as described elsewhere^49^. Briefly, SbcCD protein complex was overexpressed from SbcCD-overexpressing plasmid pDL761 in *E. coli* DL776. Cell extracts were prepared using lysis buffer (50 mM Tris-HCl, pH 7.5, 1 mM EDTA, 50 mM NaCl, 10 mM β-mercaptoethanol, 10% (w/v) sucrose, 1 mM phenylmethylsulfonyl fluoride) by sonication followed by polyethyleneimine precipitation. SbcCD was further purified by HiTrap DEAE (GE Healthcare), ammonium precipitation, Superose 6 HR 10/30 (Amersham) and Mono Q HR 5/5 (Amersham). The peak fraction was dialyzed in SbcCD strage buffer (50 mM Tris,-HCl pH 7.5, 1 mM EDTA, 50 mM NaCl, 10 mM 2–mercaptoethanol, 10% glycerol) and stored at −80 °C.

### High-speed AFM observation and image processing

Proteins in a binding buffer (25 mM Tris-HCl pH 7.5, 150 mM KCl, 2 mM MgCl_2_) were deposited on freshly cleaved mica on the glass stage with 1.5 mm diameter and incubated for 2 min before washing out unbound proteins. Proteins on mica were then visualized in an imaging buffer (25 mM Tris-HCl pH 7.5, 75 mM KCl, 3 mM MgCl_2_) using a laboratory-built high-speed AFM operated with the tapping mode. For real-time imaging of dynamic motions, proteins were directly added into the binding buffer in the imaging chamber without pre-incubation while the scanning the mica surface. Binding and imaging buffers were supplemented with ATP or ATP-γ-S at the final concentration of 1 mM when indicated.

All images were processed by using mean-flatten and pixel-average filters using a laboratory-built software based on IgorPro (Wavemetrics inc. Lake Oswego, OR). The height color scales are shown with images.

### Estimation of persistence length of MRN complex

Contour lengths along the coiled-coil arm and end-to-end distances between the ends of the coiled-coil arm were manually measured using the image-processing software mentioned above (Supplementary Fig. 2e). The averaged mean-square end-to-end distance 〈*r*^*2*^〉 for each 10% interval of the shortest contour length was plotted as a function of the averaged contour length *L*. Then the persistence length *L*_p_ was obtained by fitting the plot with the 2D WLC model as follows:

1 $$\langle r^2 \rangle = 4L_{\mathrm{p}}L\left( {1 - \frac{{2L_{\mathrm{p}}}}{L}\left( {1 - e^{ - L/L_{\mathrm{p}}}} \right)} \right)$$

### Reporting summary

Further information on research design is available in the Nature Research Reporting Summary linked to this article.

## Supplementary information

Supplementary Information

Peer Review File

Reporting Summary

Description of Additional Supplementary Files

Supplementary Movie 1

Supplementary Movie 2

Supplementary Movie 3

Supplementary Movie 4

Supplementary Movie 5

Supplementary Movie 6

Supplementary Movie 7

## Data Availability

The source data for Fig. 4e and Supplementary Figures 1a, 1b, 1c, 1e, 2c, and 3c are provided as a Source data file. All data supporting the findings of this study are available from the corresponding authors upon reasonable request.

## Source Data

Source Data File
