# Supplementary material for: Rad50 zinc hook functions as a constitutive dimerization module interchangeable with SMC hinge
Source: Nat Commun. 2020 Jan 17;11:370. doi: 10.1038/s41467-019-14025-0 (PMC6969161; doi:10.1038/s41467-019-14025-0)
Supplement: Supplementary file 1 — Supplementary Information [file 41467_2019_14025_MOESM1_ESM.pdf]

SUPPLEMENTARY INFORMATION

Rad50 zinc hook functions as a constitutive dimerization module interchangeable with SMC hinge

Hisashi Tatebe, Chew Theng Lim, Hiroki Konno, Kazuhiro Shiozaki, Akira Shinohara, Takayuki Uchihashi and Asako Furukohri

Supplementary Information List:

Supplementary Figure 1

Supplementary Figure 2

Supplementary Figure 3

Supplementary Table 1

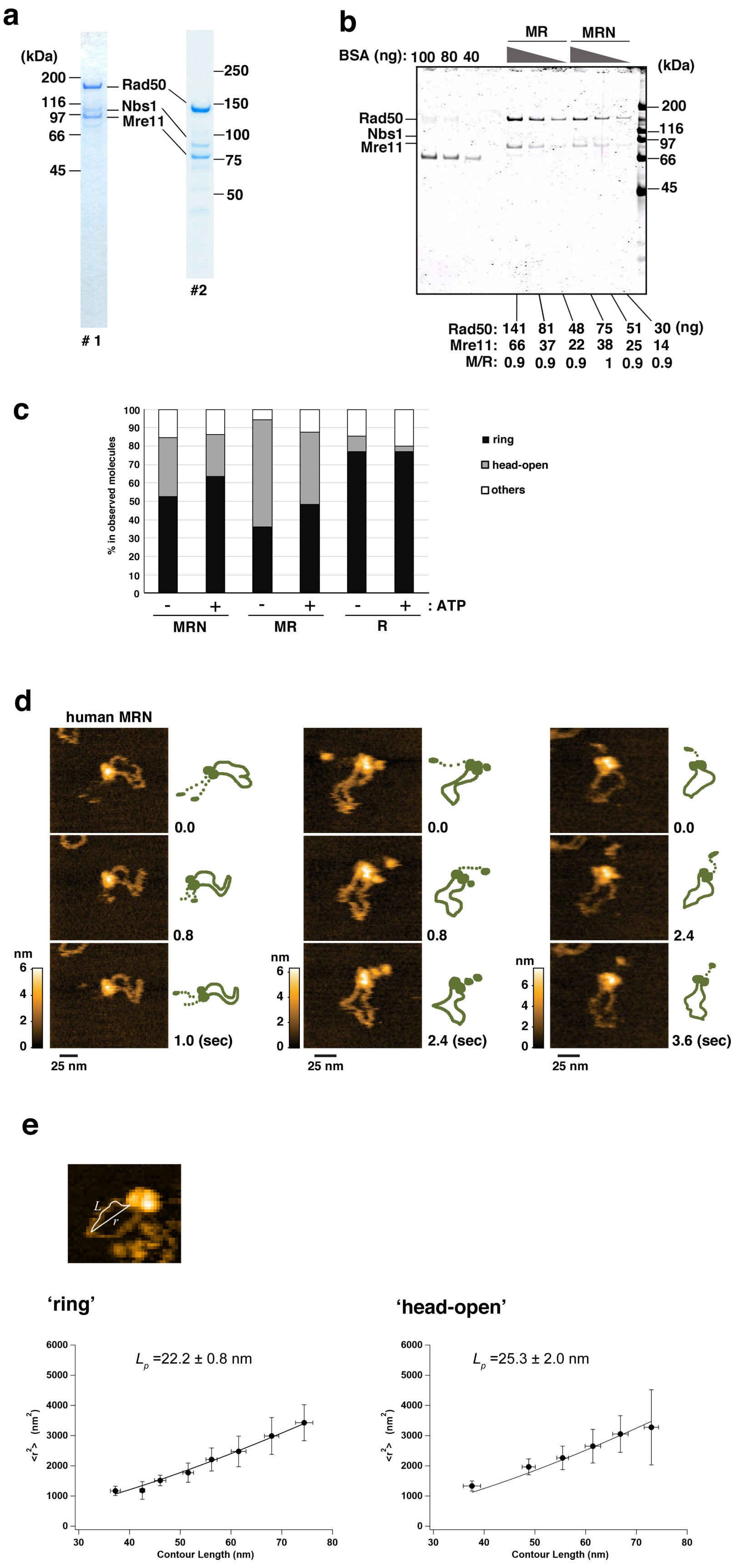

Supplementary Figure 1.

a. Purified human Mre11/Rad50/Nbs1 (MRN). Preparations #1: MRN_{His}, #2: MRN_{notag}. Proteins were separated on 4–12% NuPAGE bis-tris gel (Invitrogen) and visualized by the Colloidal Blue Staining Kit (Invitrogen) for #1, or on Supersep Ace 5-20% gel (Wako) and stained by QC colloidal Coomassie (Bio Rad) for #2. Source data are provided as a source data file.
b. Indicated amounts of BSA, human Mre11/Rad50, or MRN_{His} were separated and visualized by using a Colloidal Blue Staining Kit and an Odyssey Imaging System (LI-COR) in the 700-nm channel. The amount of protein in each band was estimated using band intensities of BSA as a standard and are shown below the image together with the ratio of Mre11/Rad50. Source data are provided as a source data file.
c. The percentages of each structure of human MRN, Mre11/Rad50 and Rad50 only are shown. Proteins were pre-incubated in Binding buffer with/without ATP for 5 min at room temperature. Proteins were then applied to mica and visualized in Imaging buffer in the presence/absence of ATP. A total of each molecules are: 221 molecules for MRN "-ATP", 194 molecules for MRN "+ATP", 153 molecules for MR "-ATP", 211 molecules for "+ATP", 195 molecules for Rad50 "-ATP" and 195 molecules for Rad50 "+ATP". Source data are provided as a source data file.
d. Small globular molecules tethered to the head domain of MRN observed in the absence of ATP. Imaging speed: 200 msec/frame.
e. The persistence length (L_p) of "ring" or "head-open" MRN. L_p is determined from contour length (l) and end-to-end distance (r). Source data are provided as a source data file.

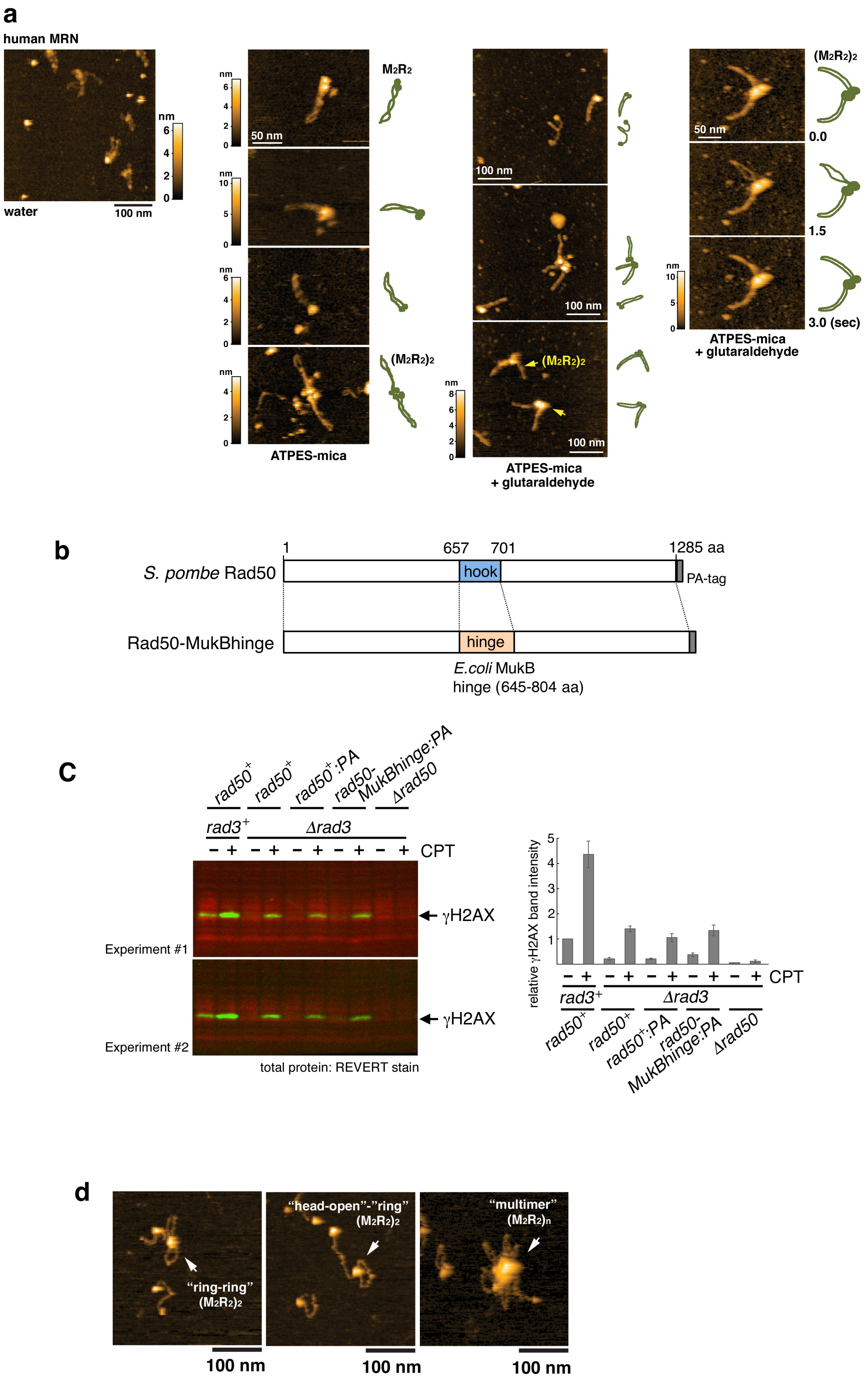

Supplementary Figure 2.

a. Human MRN observed in different conditions. Left: MRN on mica observed in water. Middle: MRN on APTES-mica observed in Imaging Buffer. Right: MRN crosslinked on APTES-mica by glutaraldehyde observed in Imaging Buffer. The presumed structures of the images are depicted next to the images.
b. Schematic representation showing *S. pombe* Rad50 and chimeric Rad50 MukBhinge.
c. γH2A levels in the presence or absence of CPT were examined in strains of the following genotypes: *rad3$^+$ rad50$^+$* (KS1598), *Δrad3 rad50$^+$* (FY32725), *Δrad3 rad50$^+$:PA* (HT1770), *Δrad3 rad50-MukBhinge:PA* (HT1768), and *Δrad3 Δrad50* (HT1782) as in Figure 4e. Crude cell extract was subjected to immunoblotting with anti-γH2A antibodies and IR-dye conjugated secondary antibodies. Signals at 800 nm (green) were detected and quantified with an Odyssey imaging system (Li-COR). Total protein on the same membrane was detected using REVERT Total Protein Stain (Li-COR) as 700-nm signals (red). γH2A band intensities were quantified and normalized to the signals of total protein between 10~20 kDa. Relative band intensities of each bands to the band of KS1598 (-CPT) were calculated. The experiments were biologically duplicated and average values were shown as a graph with SD. Source data are provided as a source data file.
d. Human Mre11/Rad50 forming intermolecular complexes. Left: the "ring"-"ring" $(M_2R_2)_2$, middle: the "head-open"-"ring" $(M_2R_2)_2$, right: the multimerized complexes are indicated by white arrowheads.

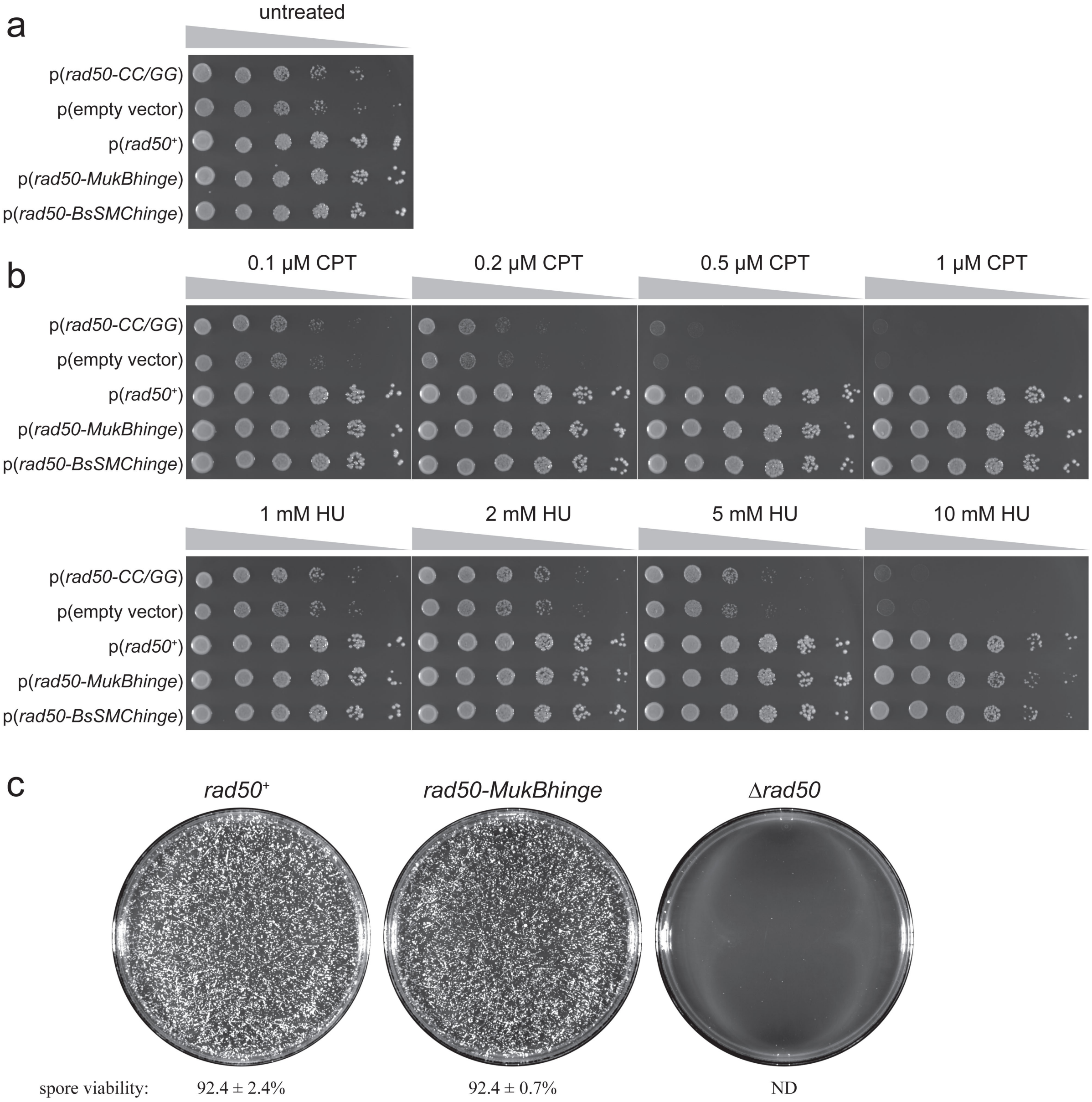

Supplementary Figure 3. The bacterial SMC hinge can substitute the fission yeast Rad50 zinc-hook *in vivo*.

a. Fission yeast Rad50 in which the zinc-hook is replaced with either the *Escherichia coli* MukB hinge or the *Bacillus subtilis* SMC hinge rescues slow growth phenotypes of the Δ*rad50* mutant in untreated condition. The Δ*rad50* mutant strain (HT1250) was transformed with an "empty vector" control or *rad50* plasmids carrying either the wild-type *rad50* ("*rad50*$^{+}$") allele, the "*rad50-CC/GG"* allele in which the two essential cysteine residues Cys674 and Cys677 of the zinc-hook

were replaced with glycine, the "*rad50-MukBhinge"* allele in which the zinc-hook (657-701 aa) was replaced with the *E. coli* MukB hinge (645-804 aa), or the *"rad50-BsSMChinge"* allele in which the zinc-hook was replaced with the *B. subtilis* SMC hinge (487-684 aa). These transformants were grown in the EMM liquid medium at 30°C, after which 5-fold serial dilutions were onto EMM agar. Photos were taken after 3-day incubation.

b. Fission yeast Rad50 carrying the *Ec* MukB hinge or the *Bs* SMC hinge instead of the zinc-hook rescues severe sensitivities of the Δ*rad50* mutant to camptothecin (CPT) and hydroxyurea (HU). Sensitivities to CPT and HU were examined as in (a). The EMM solid medium contained the indicated amounts of CPT or HU. Photos were taken after 3-day or 5-day incubation.

c. *rad50-MukBhinge* homozygotes produce viable spores. Twenty thousand spores shown in Figure 4f were spread onto YES agar and incubated. Spore viability was measured as described in Materials and Methods. Photos were taken after 2-day incubation. Source data are provided as a source data file.

Supplementary Table 1. Fission yeast strains used in this study.

Strain ID	Genotype	Source, Reference
KS1598	*h⁻ leu1-32*	Lab stock
PR109	*h⁻ leu1-32 ura4-D18*	Lab stock
CHP428	*h⁺ leu1-32 ura4-D18 his7-366 ade6-M210*	Lab stock
FY32724	*h⁻ leu1-32 ura4-D18 rad52:YFP(kanMX6)*	NBRP Yeast
FY32725	*h⁻ leu1-32 ura4-D18 Δrad3::ura4⁺*	NBRP Yeast
HT1250	*h⁻ leu1-32 Δrad50::kanMX6*	This study
HT1257	*h⁺ leu1-32 his7-366 Δrad50::kanMX6*	This study
HT1633	*h⁻ leu1-32 rad50⁺:PA(kanMX6)*	This study
HT1655	*h⁺ leu1-32 rad50-MukBhinge:PA(kanMX6)*	This study
HT1680	*h⁺ leu1-32 his7-366 rad50-MukBhinge:PA(kanMX6)*	This study
HT1683	*h⁻ leu1-32 rad50-MukBhinge:PA(kanMX6)*	This study
HT1693	*h⁺ leu1-32 his7-366 rad50⁺:PA(kanMX6)*	This study
HT1697	*h⁻ leu1-32 rad50⁺:PA(kanMX6)*	This study
HT1768	*h⁻ leu1-32 ura4-D18 Δrad3::ura4⁺ rad50-MukBhinge:PA(kanMX6)*	This study
HT1770	*h⁻ leu1-32 ura4-D18 Δrad3::ura4⁺ rad50:PA(kanMX6)*	This study
HT1782	*h⁺ leu1-32 ura4-D18 Δrad3::ura4⁺ Δrad50::kanMX6*	This study
HT1790	*h⁻ leu1-32 rad52:YFP(kanMX6) Δrad50::kanMX6*	This study
HT1791	*h⁻ leu1-32 rad52:YFP(kanMX6) rad50-MukBhinge:PA(kanMX6)*	This study
HT1792	*h⁻ leu1-32 rad52:YFP(kanMX6) rad50:PA(kanMX6)*	This study
