# Supplementary material for: Rad50 zinc hook functions as a constitutive dimerization module interchangeable with SMC hinge
Source: Nat Commun. 2020 Jan 17;11:370. doi: 10.1038/s41467-019-14025-0 (PMC6969161; doi:10.1038/s41467-019-14025-0)
Supplement: Supplementary file 3 — Reporting Summary [file 41467_2019_14025_MOESM3_ESM.pdf]

nature research

Corresponding author(s): Asako Furukohri

Last updated by author(s): Nov 29, 2019

Reporting Summary

Nature Research wishes to improve the reproducibility of the work that we publish. This form provides structure for consistency and transparency in reporting. For further information on Nature Research policies, see Authors & Referees and the Editorial Policy Checklist.

Statistics

For all statistical analyses, confirm that the following items are present in the figure legend, table legend, main text, or Methods section.

n/a	Confirmed	
☐	☒	The exact sample size (*n*) for each experimental group/condition, given as a discrete number and unit of measurement
☐	☒	A statement on whether measurements were taken from distinct samples or whether the same sample was measured repeatedly
☒	☐	The statistical test(s) used AND whether they are one- or two-sided<br>*Only common tests should be described solely by name; describe more complex techniques in the Methods section.*
☒	☐	A description of all covariates tested
☒	☐	A description of any assumptions or corrections, such as tests of normality and adjustment for multiple comparisons
☐	☒	A full description of the statistical parameters including central tendency (e.g. means) or other basic estimates (e.g. regression coefficient) AND variation (e.g. standard deviation) or associated estimates of uncertainty (e.g. confidence intervals)
☒	☐	For null hypothesis testing, the test statistic (e.g. *F*, *t*, *r*) with confidence intervals, effect sizes, degrees of freedom and *P* value noted<br>*Give P values as exact values whenever suitable.*
☒	☐	For Bayesian analysis, information on the choice of priors and Markov chain Monte Carlo settings
☒	☐	For hierarchical and complex designs, identification of the appropriate level for tests and full reporting of outcomes
☒	☐	Estimates of effect sizes (e.g. Cohen's *d*, Pearson's *r*), indicating how they were calculated

Our web collection on statistics for biologists contains articles on many of the points above.

Software and code

Policy information about availability of computer code

Data collection	High-speed AFM images were collected using a laboratory-built software developed based on on IgorPro ver. 6 (WaveMietrics Inc. Lakse Oswego, OR)
Data analysis	High-speed AFM experiments and image analysis were carried out using a laboratory-built software developed based on on IgorPro ver. 6 (WaveMietrics Inc. Lakse Oswego, OR). Western blotting signals were processed using Image Quant TL (GE healthcare), Odyssay Application Software ver. 1.2 (Li-COR) and Photoshop Elements (Adobe).

For manuscripts utilizing custom algorithms or software that are central to the research but not yet described in published literature, software must be made available to editors/reviewers. We strongly encourage code deposition in a community repository (e.g. GitHub). See the Nature Research guidelines for submitting code & software for further information.

Data

Policy information about availability of data

All manuscripts must include a data availability statement. This statement should provide the following information, where applicable:

- Accession codes, unique identifiers, or web links for publicly available datasets
- A list of figures that have associated raw data
- A description of any restrictions on data availability

The data that support the findings of this study are available from the corresponding author (A.F.) upon reasonable request. The source data for Fig. 4e and Supplementary Figs S1a, S1b, S1c, S1e, S2c and S3c are provided as a Source Data File.

Field-specific reporting

Please select the one below that is the best fit for your research. If you are not sure, read the appropriate sections before making your selection.

☒ Life sciences ☐ Behavioural & social sciences ☐ Ecological, evolutionary & environmental sciences

For a reference copy of the document with all sections, see nature.com/documents/nr-reporting-summary-flat.pdf

Life sciences study design

All studies must disclose on these points even when the disclosure is negative.

Sample size	Sample size was decided on the basis of previous experience to evaluate the reproducibility. No statistical methods were used to determine the sample size.
Data exclusions	For counting the number of each structures in HS-AFM images, we excluded images that contain too many molecules because we cannot recognize each molecules. In addition, we excluded molecules that are obviously aggregated or broken under the observation.
Replication	HS-AFM images were representative obtained by repeated experiments. Main data (structures of human MRN and Mre11/Rad50) were determined using two independently purified protein samples. For biological analysis, at least two independently obtained mutant strains were analyzed to reach conclusions.
Randomization	Not applicable to this study because group allocation was not carried out.
Blinding	Not applicable to this study because group allocation was not carried out.

Reporting for specific materials, systems and methods

We require information from authors about some types of materials, experimental systems and methods used in many studies. Here, indicate whether each material, system or method listed is relevant to your study. If you are not sure if a list item applies to your research, read the appropriate section before selecting a response.

Materials & experimental systems

n/a	Involved in the study	
☐	☒	Antibodies
☒	☐	Eukaryotic cell lines
☒	☐	Palaeontology
☒	☐	Animals and other organisms
☒	☐	Human research participants
☒	☐	Clinical data

Methods

n/a	Involved in the study	
☒	☐	ChIP-seq
☒	☐	Flow cytometry
☒	☐	MRI-based neuroimaging

Antibodies

Antibodies used	Antibodies: anti-phospho H2A (S129) antibodies (Abcam, ab17353), goat anti-rabbit IgG IRDye 800CW conjugated (Abcam, ab216773), rat anti-PA tag antibody (Wako, 012-25863), donkey anti-rat IgG antibody (Jackson, 712-035-150) were used for western blotting analysis.
Validation	Validations are based on information provided from the manufactures.
