# Supplementary material for: Rad50 zinc hook functions as a constitutive dimerization module interchangeable with SMC hinge
Source: Nat Commun. 2020 Jan 17;11:370. doi: 10.1038/s41467-019-14025-0 (PMC6969161; doi:10.1038/s41467-019-14025-0)
Supplement: Supplementary file 4 — Description of Additional Supplementary Files [file 41467_2019_14025_MOESM4_ESM.docx]

**Description of Additional Supplementary Files**

File name: Supplementary movie 1

Description: Dynamic structure of ring-shaped human MRN in buffer (+ATP-γ-S). Imaging speed: 130 msec per frame.

File name: Supplementary movie 2

Human MRN visualized in buffer (-ATP). Imaging speed: 590 msec per frame.

File name: Supplementary movie 3

Description: Small globular molecules tethered to the head of human MRN visualized in buffer (+ATP). Imaging speed: 150 msec per frame.

File name: Supplementary movie 4

Description: The hook-deficient mutant Mre11/Rad50 (MR_CC/GG_) visualized in bffer (-ATP). Imaging speed: 200 msec per frame.

File name: Supplementary movie 5

Description: The ring-shaped human Mre11/Rad50 visualized in buffer (-ATP). Imaging speed: 200 msec per frame.

File name: Supplementary movie 6

Description: The ring-shaped human Mre11/Rad50 visualized in buffer (-ATP). Imaging speed: 200 msec per frame.

File name: Supplementary movie 7

Description: *E. coli* Mre11/Rad50 (SbcCD) visualized in buffer (-ATP). Imaging speed:150 msec per frame.
