# Supplementary material for: Rad50 zinc hook functions as a constitutive dimerization module interchangeable with SMC hinge
Source: Nat Commun. 2020 Jan 17;11:370. doi: 10.1038/s41467-019-14025-0 (PMC6969161; doi:10.1038/s41467-019-14025-0)
Supplement: Supplementary file 2 — Peer Review File [file 41467_2019_14025_MOESM2_ESM.pdf]

Reviewers' comments:

Reviewer #1 (Remarks to the Author):

The manuscript 'Rad50 zinc hook is structurally and functionally interchangeable with SMC hinge' by Tatebe et al. reports a high-speed atomic force microscopy (HS-AFM) study of the SMC family protein Mre11/Rad50. Novel evidence is shown that Mre11/Rad50 (from bacteria, yeast and human) display structural dynamics in the head domains and not in the hinge/hook domains as previously reported.

Mre11/Rad50 is involved in genome maintenance, ie double strand break repair, thus of biomedical and biotechnological significance. Further evidence is shown that the 'zinc-hook' present in Rad50 corresponds to the 'SMC-hinge' in other SMC proteins, as chimeric proteins of these domains are functionally quasi indistinguishable.

The authors analyze the complex from various species, bacteria, yeast and human, and chimeric complexes, and show compelling data that the head-domains open the ring-like arrangement. Previously, it has been suggested, based on much less compelling data than shown here, that the hinge (or hook in some species) domain on the opposite side of the complex dissociates. This data has been functionally interpreted with rather important implications. Thus this paper has tremendous importance setting this straight. In this context it is somewhat regretful that the writing is not always at the level of the data, and, especially in context of the debate about the structural dynamics, the wording is sometimes vague and evasive. This reviewer even thinks that the fact that the structural dynamics are situated in the head-domain should be in the title. This seems to me like a much more important message than the fact that the Rad50 zinc hook is interchangeable with the coli hinge.

My detailed observations below:

abstract: define the previous 'nanoscale imaging'

Introduction and Figure 1: The Mre11/Rad50 complex has M2R2 stoichiometry. Figure 1a is unfortunate, as it (i) does not precisely give the reader the opportunity to understand which parts of the complex are Mre11 and which ones Rad50, and (ii) the color coding of the schematic in 1a (left) seems to suggest that the stoichiometry was M2R4. Please amend and clarify.

Introduction page 3 line 22 to page 4 line 4: This entire paragraph is unclear. It seems like the authors think that some previous AFM studies presented artefacts but avoid to say or discuss really what they think. The authors contort their sentences trying to avoid a clear message which is regretful because it weakens their own work. References are grouped, eg. (1,3-7), to an extend that one does not know which reference stands for what; in contrast the key sentence "On the other hand, previous studies to visualize the entire architecture of the complex by atomic force microscopy (AFM) showed..." has no reference! Please amend and clarify. There are other sections of the paper talking about the discrepancy of the presented and former data that could also be improved. It seems to this reviewer the most important aspect of the paper, because the authors of the previous works drew rather far-reaching conclusions based on their findings.

Introduction page 4 line 13: 'corroborated' instead of 'collaborated'?

Results page 5: The flexibility of the arms is often qualitatively described. I suggest the authors measure the persistence length and quantify the arms' properties.

Results page 5: Is it possible to adjust the imaging conditions to better immobilize the Nbs1 domains? (This comment is not compulsory)

Page 5 and figure 1f: The hook mutant clearly shows that the head 'forces' the arms to spread ('arms of the Mre11/Rad50CC/GG complex protruded from the globular domain in opposite directions'). This apparent bending rigidity should mean that the head-domain drives the structure of the entire complex towards a circle in equilibrium, right?

Results, page 6 and figure 2: The authors make a rather strong case about the arms and the identification of a possible elbow-like structure approximately central along the arm. I have trouble following the authors on this argument for the following reasons: (1) The 2D adsorption of the molecules on mica, may represent apparent kinks in flexible domains that are difficult to understand what they originate from. (2) in figure 2c panels iii and iv (and to a lesser extend ii) I could imagine the strands cross, because I see the putative elbows in contact. (3) Is there a sequence in the arm domain that would indicate some sort of a weakened stretch that could become the elbow? (4) What would a physical model predict? If one adsorbs a flexible rod-like structure on a 2d plane with confined ends, wouldn't the physics predict that buckling occurs midway? (5) I don't see any signature of an elbow in 2d, shouldn't I be able to also see the elbow in these 'softer conditions'.

results, page 7 around line 20: Finally the authors take a more clear statement that the former studies showing hook-open structures were artifactual. The writing can however be improved here, as the authors first say about their own work that they saw 'rarely' such configurations before rejecting these observations as 'illusory' (which is an unfortunate choice of wording), then giving good arguments that this is not the case. I think the strongest argument was provided in their mutant experiment (fig 1f) where they show that the head drives the ends of the arms far apart when the hook is undone.

page 8 line 5: what do the authors mean by 'although the coiled-coil arms of yeast Mre11/Rad50 are more crooked like elbows'

The data is of highest quality and the figures well-done.

Reviewer #2 (Remarks to the Author):

The manuscript presents a high-speed atomic force microscopy (HS-AFM) characterization of Mre11/Rad50 (M2R2) DNA repair complexes from several organisms. Previous studies of these complexes used conventional AFM. The authors conduct a detailed revision of the previous studies and conclude that the conformational features of Mre11/Rad50 are conserved across species and are similar to those of the SMC proteins. This is a welcome but not really an unexpected result. The main innovation of the paper is the construction of a chimeric Mre11/Rad50 complex containing the hinge domain from an SMC protein, MukB, instead of the Rad50 zinc-hook. Confusingly, the authors refer to MukB as SMC, in abstract and the main text. MukB is a divergent member of the SMC family, the protein must be named explicitly. The authors report that the chimera is functionally active, which offers grounds for mechanistic speculations.
Specific concerns:
1. Previous studies revealed that one of the major shapes for the human and yeast complexes is "hook-open" M2R2 connected only via the head, but such a "hook-open" molecule is not observed with the archaeal and bacterial complexes. Authors of current paper did not find "hook-open" structure in any of the analyzed proteins. They speculated that the reason of such structure could be denaturation of proteins when they are tightly fixed on mica in conventional AFM. This hypothesis could be addressed within HS-AFM technique. Authors admitted observations of "images showing disconnection" of coiled-coil/hook/head "that caused illusory" (page 7). What if it is not "illusory" but reflects a fast dissociation/association of the hook domains? Is it possible that tight protein binding to mica captured dissociation step of human and yeast Mre11/Rad50? Another possibility to address is in experimental differences between the previous and the current studies (local temperature, timing, etc)
2. The "hook-open" conformation of human and yeast Mre11/Rad50 was found mostly in the

complexes with DNA, although free proteins were also able to show such a conformation. It is disappointing that the current manuscript does not have experiments with DNA.
3. The authors claim that Mre11 and Nbs1 subunits do not affect much the structural features of Rad50 (Page 5-6). This result must be statistically analyzed for all presented structures as it was done in figure S1C for experiment with and without ATP.
4. The manuscript confirms the previous finding that ATP binding increases the percentage of closed rings in the Mre11/Rad50 complex. Is it true for Rad50 alone?
5. The manuscript has beautiful pictures and a movie with a small molecule attached to Rad50 when Mre11/Rad50/Nbs1 protein prep was visualized. The authors speculate that this molecule is Nbs1. Can the authors substantiate this claim?

Reviewer #3 (Remarks to the Author):

Tatebe et al report their studies of the MRN protein complex that is required for DSB repair, focusing on the zinc-hook in Rad50, which is a dimerization interface. Combining HS-AFM with yeast genetics experiments, they make a compelling case for the Rad50 zinc-hook functioning as a stable dimerization interface that does not need to open in order for the MRN complex to fulfill its DNA repair functions, akin to the hinge in SMC proteins. In fact, part of their evidence involves the replacement of the zinc-hook with an SMC hinge. These yeast studies were performed to a high standard. Taken as a whole, these experiments provide persuasive answers about the structural requirements for the MRN complex.

Two minor suggestions:

1. Based on the data I would not characterize the rad50 null growth defect as ‘modest’. It seems greater than that.
2. Although the chimera constructions are uncomplicated, for some readers it still might be helpful to see the gene constructions illustrated in a figure, perhaps in the supplement.

[Point-by-point responses]

First of all, we thank all reviewers for their constructive evaluations. We believe that the revised manuscript has been greatly improved according to their helpful comments.
Reviewers' comments (in the black font) and author's responses (in the red font) are as below:
(Page and line numbers refer to locations in the revised manuscript)

Reviewer #1 (Remarks to the Author):

The manuscript 'Rad50 zinc hook is structurally and functionally interchangeable with SMC hinge' by Tatebe et al. reports a high-speed atomic force microscopy (HS-AFM) study of the SMC family protein Mre11/Rad50. Novel evidence is shown that Mre11/Rad50 (from bacteria, yeast and human) display structural dynamics in the head domains and not in the hinge/hook domains as previously reported.

Mre11/Rad50 is involved in genome maintenance, ie double strand break repair, thus of biomedical and biotechnological significance. Further evidence is shown that the 'zinc-hook' present in Rad50 corresponds to the 'SMC-hinge' in other SMC proteins, as chimeric proteins of these domains are functionally quasi indistinguishable.

The authors analyze the complex from various species, bacteria, yeast and human, and chimeric complexes, and show compelling data that the head-domains open the ring-like arrangement. Previously, it has been suggested, based on much less compelling data than shown here, that the hinge (or hook in some species) domain on the opposite side of the complex dissociates. This data has been functionally interpreted with rather important implications. Thus this paper has tremendous importance setting this straight. In this context it is somewhat regretful that the writing is not always at the level of the data, and, especially in context of the debate about the structural dynamics, the wording is sometimes vague and evasive. This reviewer even thinks that the fact that the structural dynamics are situated in the head-domain should be in the title. This seems to me like a much more important message than the fact that the Rad50 zinc hook is interchangeable with the coli hinge.

[Author's response 1-1]
We thank Referee #1 for his/her deep understanding of the importance of our findings. We recognized that the referee encouraged us to state the main conclusion more clearly by directly comparing previous observations and ours. We have substantially modified the main text accordingly (page 3, line 17-23, line 26-28; page 4, line 1-4). In addition, we have carried out new experiments in response to the referee's comments, and the results suggest the possible cause of the discrepancy between the previous AFM reports and our observation (see [Author's response 2-1]). We have included these new data and speculations in the main text (page 7, line 27-33; page 8, line 1-21, Supplementary Figure S2a).

Referee #1 also suggested that the title indicate that the structural dynamics are in the head domain. We agree that the previous title did not precisely describe our findings, which is that MRN persistently closes the ring at the hook, while occasionally opens it at the head. Although these two expressions describe the same molecule's dynamics, we prefer the former one in the title because in the latter part of the manuscript we focus on the hook, not on the head. Our *in vivo* data suggests that opening the ring at the hook is unlikely. The new title is 'Rad50 zinc hook functions as a constitutive dimerization module interchangeable with SMC hinge'. We hope you agree that the new title is more appropriate than the previous one.

My detailed observations below:

abstract: define the previous 'nanoscale imaging'

[Author's response 1-2]

In response to this comment, we have slightly modified the relevant sentence (page 2, line 4-5). The abstract should be unreferenced and shortened for formatting in the journal's style. Thus, we also modified resemble sentences in the main text to define how previous observations were obtained (page 3, line 17-23), as explained in [Author's response 1-4].

Introduction and Figure 1: The Mre11/Rad50 complex has M2R2 stoichiometry. Figure 1a is unfortunate, as it (i) does not precisely give the reader the opportunity to understand which parts of the complex are Mre11 and which ones Rad50, and (ii) the color coding of the schematic in 1a (left) seems to suggest that the stoichiometry was M2R4. Please amend and clarify.

[Author's response 1-3]

We agree that the previous cartoon did not clearly show M2R2 stoichiometry. We have modified it according to the referee's comments in Figure 1a (left) in the revised manuscript.

Introduction page 3 line 22 to page 4 line 4: This entire paragraph is unclear. It seems like the authors think that some previous AFM studies presented artefacts but avoid to say or discuss really what they think. The authors contort their sentences trying to avoid a clear message which is regretful because it weakens their own work. References are grouped, eg. (1,3-7), to an extend that one does not know which reference stands for what; in contrast the key sentence "On the other hand, previous studies to visualize the entire architecture of the complex by atomic force microscopy (AFM) showed..." has no reference! Please amend and clarify.

[Author's response 1-4]

According to the referee's comment, we have modified these unclear sentences (page 3, line 17-28). The previous observations have been described and referenced individually. The previous AFM observation in solution, which we compared with our result, has been individually described with an appropriate reference in the revised manuscript.

There are other sections of the paper talking about the discrepancy of the presented and former data that could also be improved. It seems to this reviewer the most important aspect of the paper,

because the authors of the previous works drew rather far-reaching conclusions based on their findings.

[Author's response 1-5]

We have modified the main sentences according to the referee's comment (page 7, line 27-33; page 8, line 1-21), as mentioned in [Author's response 1-1] and [Author's response 1-11].

eResults page 5: Is it possible to adjust the imaging conditions to better immobilize the Nbs1 domains? (This comment is not compulsory)

[Author's response 1-8]

In response to this comment, we have tested several conditions intended to immobilize the Nbs1 subunit. Unexpectedly, we found that a tight fixation of MRN to a mica surface somehow causes denaturation of the protein structure as mentioned in [Author's response 2-1], so it is difficult to immobilize Nbs1 on mica to observe it in a better condition.

Instead, we have purified another preparation of MRN protein (no tagged Nbs1) as in Supplementary Figure S1a, which contains slightly more Nbs1 subunits than the previous MRN preparation (His-tagged Nbs1). Using this preparation, we again observed that small, oval structures connected with over 20-nm long, string-like structures to the head domains of MRN, which were not observed with Mre11/Rad50 only. We observed several types of such structures (Supplementary Figure S1d), but we could not carry out a statistical analysis because those structures were still hardly ever observed. Because it is currently difficult for us to provide further evidence, we have modified the main text to clearly describe that it is just one of the possible explanations of what the observed structure is (page 5, line 7-12).

Page 5 and figure 1f: The hook mutant clearly shows that the head 'forces' the arms to spread ('arms of the Mre11/Rad50CC/GG complex protruded from the globular domain in opposite directions'). This apparent bending rigidity should mean that the head-domain drives the structure of the entire complex towards a circle in equilibrium, right?

[Author's response 1-9]

We agree that the head seems to force the arms to spread (as shown in Figure 2c-i and 2c-iv), suggesting that the structural changing of the head can affect the entire structure of the complex. We think that when the head forces the arms to spread in opposite directions while the hook tightly connects the curved arms, the entire complex becomes a circle, as Referee #1 pointed out. If the head structure is changed, the protruded arms can be parallel and the circle becomes a crescent-like shape ("closed/arm-parallel" structure) as observed in Figure 2c-ii. In response to this comment, we have slightly modified the main text (page 5, line 26-27).

Results, page 6 and figure 2: The authors make a rather strong case about the arms and the identification of a possible elbow-like structure approximately central along the arm. I have trouble following the authors on this argument for the following reasons: (1) The 2D adsorption of the molecules on mica, may represent apparent kinks in flexible domains that are difficult to understand what they originate from. (2) in figure 2c panels iii and iv (and to a lesser extend ii) I

could imagine the strands cross, because I see the putative elbows in contact. (3) Is there a sequence in the arm domain that would indicate some sort of a weakened stretch that could become the elbow? (4) What would a physical model predict? If one adsorbs a flexible rod-like structure on a 2d plane with confined ends, wouldn't the physics predict that buckling occurs midway? (5) I don't see any signature of an elbow in 2d, shouldn't I be able to also see the elbow in these 'softer conditions'.

[Author’s response 1-10]

As the referee pointed out, the description of the elbow-like structure may be too strong. We have deleted most of the description of the elbow-like structure from the revised manuscript and marks in Figure 1b, 1e and 3a. The entire section is modified accordingly (page 6, line 21-27).

Detailed answers are as below:

(1, 3, 4) As the referee pointed out, it is possible that 2D adsorption of the protein on mica may force the arm to kink at a particular position, so we have included this possibility in the main text (page 6, line 21-27) because it is reported that coiled-coil free regions exist in the middle of arms and this region is predicted to be more flexible than coiled-coil regions as mentioned in the main text (page 6, line 28-29). (2) Although it is difficult to recognize, the coiled-coil arms seem not to be crossed in the movie (Supplementary movie S5 and S6). (5) When Mre11/Rad50 does not tightly bind to a mica surface (as in Figure 2d), it is difficult to recognize the elbow-like structure because the entire arms flexibly move so quickly.

results, page 7 around line 20: Finally the authors take a more clear statement that the former studies showing hook-open structures were artifactual. The writing can however be improved here, as the authors first say about their own work that they saw 'rarely' such configurations before rejecting these observations as 'illusory' (which is an unfortunate choice of wording), then giving good arguments that this is not the case. I think the strongest argument was provided in their mutant experiment (fig 1f) where they show that the head drives the ends of the arms far apart when the hook is undone.

[Author’s response 1-11]

According to the referee’s comment, we have modified the main text to improve the writing (page 7, line 27-33; page 8, line 1-21). In addition, to describe data more precisely, the actual number of the ‘hook-open’-like structure we observed is now shown in the main text (page 5, line 18-19, less than 1%). We have included the discussion about the mutant experiment as suggested by the referee (page 8, line 20) and have explained why the observed structures could different between the previous observations and our observations as mentioned earlier in [Author’s response 1-1] and later in [Author’s response 2-1].

page 8 line 5: what do the authors mean by 'although the coiled-coil arms of yeast Mre11/Rad50 are more crooked like elbows'

[Author’s response 1-12]

We occasionally observed a crooked, distinguishable structure at a specific location of the arms of yeast Mre11/Rad50 as observed in Figure 3a-i. However, it may not be so clear and we did not

carry out any statistical/quantitative analysis about this structure. So we removed most of description of the 'elbow' from the main manuscript as mentioned earlier in [Author's response 1-10].

The data is of highest quality and the figures well-done.

Reviewer #2 (Remarks to the Author):

The manuscript presents a high-speed atomic force microscopy (HS-AFM) characterization of Mre11/Rad50 (M2R2) DNA repair complexes from several organisms. Previous studies of these complexes used conventional AFM. The authors conduct a detailed revision of the previous studies and conclude that the conformational features of Mre11/Rad50 are conserved across species and are similar to those of the SMC proteins. This is a welcome but not really an unexpected result. The main innovation of the paper is the construction of a chimeric Mre11/Rad50 complex containing the hinge domain from an SMC protein, MukB, instead of the Rad50 zinc-hook. Confusingly, the authors refer to MukB as SMC, in abstract and the main text. MukB is a divergent member of the SMC family, the protein must be named explicitly. The authors report that the chimera is functionally active, which offers grounds for mechanistic speculations.
[Author's response to this comment]
In response to this comment, we have modified the abstract and changed the words from 'SMC hinge' to 'the SMC hinge of bacterial condensin MukB' in both the abstract and the main text (page 2, line 12-13; page 4, line 8).

Specific concerns:
1. Previous studies revealed that one of the major shapes for the human and yeast complexes is "hook-open" M2R2 connected only via the head, but such a "hook-open" molecule is not observed with the archaeal and bacterial complexes. Authors of current paper did not find "hook-open" structure in any of the analyzed proteins. They speculated that the reason of such structure could be denaturation of proteins when they are tightly fixed on mica in conventional AFM. This hypothesis could be addressed within HS-AFM technique. Authors admitted observations of "images showing disconnection" of coiled-coil/hook/head "that caused illusory" (page 7). What if it is not "illusory" but reflects a fast dissociation/association of the hook domains? Is it possible that tight protein binding to mica captured dissociation step of human and yeast Mre11/Rad50? Another possibility to address is in experimental differences between the previous and the current studies (local temperature, timing, etc)
[Author's response 2-1]
According to the referee's comment, we have modified the main text (page 7, line 27-33; page 8, line 1-21). In addition, Referee #2 pointed out the possibility that in fact Rad50 disconnects the hook in "images showing disconnection". He/she also asked if it is possible that a tight protein binding to mica would allow us to capture this dissociation step. In response to this and Referee

#1's comment, we have tried several conditions to tightly immobilize the complex. In the previous work using a conventional AFM, MRN was once deposited on mica, washed with water and then air-dried before the observation. We found that the structure of MRN was entirely destroyed by exposure of the complex to water (Supplementary Figure S2a, left), suggesting that the previous observation condition may not be appropriate.

Next, we used an aminosilane-coated mica (APTES-mica) to strongly fix MRN complex on mica. We observed that the entire structure was largely affected by the fixation on mica. As shown in Supplementary Figure S2a, middle, the entire coiled-coil arms in one molecule were attached to each other on the APTES-mica. In addition, the head domains tend to be dimerized or multimerized. This dimerized MRN ($(M_2R_2N_2)_2$ complex) was more frequently observed when we tightly crosslinked the complex on the APTES-mica by using glutaraldehyde (Supplementary Figure S2a, right).

Interestingly, this $(M_2R_2N_2)_2$ complex somehow resembles to the so-cold 'hook-open' structure reported in the previous result. In the previous experiment in 2005, the scan speed (39 seconds per frame) was ~400 times slower than our scan speed (100 milliseconds per frame). Under such a low scanning speed, a quickly moving MRN or a quickly moving subunit probably just cannot be observed. Thus, one possible explanation is that in the previous report, only the molecules tightly fixed and deformed on mica were observed and interpreted incorrectly. We included these new data in the revised manuscript and modified the text accordingly (page 7, line 28-33; page 8, line 1-22).

2. The "hook-open" conformation of human and yeast Mre11/Rad50 was found mostly in the complexes with DNA, although free proteins were also able to show such a conformation. It is disappointing that the current manuscript does not have experiments with DNA.

[Author's response 2-2]

As the referee pointed out, observing MRN bound to DNA is important if we are to understand MRN's functions *in vivo*. However, observing DNA-MRN complexes is currently technically difficult. We tried to observe DNA-MRN complexes, but at a relatively high protein/DNA concentration suitable for observing MRN's DNA binding, it is difficult to distinguish if MRN binds to DNA or both MRN and DNA are accidentally co-located together. Previous observation of an MRN-DNA complex did not overcome this problem and the obtained image was of such low resolution that was difficult to conclude that DNA binding induces the hook-opening of MRN (Ref 14). Even for other SMC family proteins such as condensin and cohesin, which are known to bind to DNA more stably than MRN does, observing the molecular structure by high-speed AFM when they bind to DNA in solution has not been reported yet.

3. The authors claim that Mre11 and Nbs1 subunits do not affect much the structural features of Rad50 (Page 5-6). This result must be statistically analyzed for all presented structures as it was done in figure S1C for experiment with and without ATP.

[Author's response 2-3]

According to the referee's suggestion, we have statistically analyzed if the presence of Mre11 or Nbs1 affects the structural features of Rad50 (Supplementary Figure S1c). As mentioned in the previous manuscript, the statistical analysis showed that the ratio of the head-open structure of Mre11/Rad50 is higher than that of Rad50 only, suggesting that the presence of Mre11 may induce opening the ring to some extent. In addition, comparison between MRN and Mre11/Rad50 revealed that the ratio of ring-shaped architecture increases when Nbs1 is present. We included these observations in the revised manuscript and modified the main text accordingly (page 6, line 3-5, line 13-15).

4. The manuscript confirms the previous finding that ATP binding increases the percentage of closed rings in the Mre11/Rad50 complex. Is it true for Rad50 alone?

[Author's response 2-4]

We have carried out the statistical analysis as the referee suggested (Supplementary Figure S1c). As mentioned earlier in [Author's response 2-3], most of Rad50 takes a ring-like architecture even in the absence of ATP, so that it is difficult to tell whether or not the binding of ATP affects the structure of Rad50 only.

5. The manuscript has beautiful pictures and a movie with a small molecule attached to Rad50 when Mre11/Rad50/Nbs1 protein prep was visualized. The authors speculate that this molecule is Nbs1. Can the authors substantiate this claim?

[Author's response 2-5]

As mentioned earlier in [Author's response 1-8], it is currently difficult for us to provide further evidence that the observed structure is the Nbs1 subunit because it is difficult to immobilize Nbs1 on mica to observe it in a better condition. We have modified the main text to clearly describe that it is just one of the possibilities to explain what the observed structure is (page 5, line 7-12).

Reviewer #3 (Remarks to the Author):

Tatebe et al report their studies of the MRN protein complex that is required for DSB repair, focusing on the zinc-hook in Rad50, which is a dimerization interface. Combining HS-AFM with yeast genetics experiments, they make a compelling case for the Rad50 zinc-hook functioning as a stable dimerization interface that does not need to open in order for the MRN complex to fulfill its DNA repair functions, akin to the hinge in SMC proteins. In fact, part of their evidence involves the replacement of the zinc-hook with an SMC hinge. These yeast studies were performed to a high standard. Taken as a whole, these experiments provide persuasive answers about the structural requirements for the MRN complex.

Two minor suggestions:

1. Based on the data I would not characterize the rad50 null growth defect as 'modest'. It seems greater than that.

[Author's response 3-1]
We have accordingly modified the main text to characterize the growth defect of rad50 null strain as 'apparent' (page 10, line 21).

2. Although the chimera constructions are uncomplicated, for some readers it still might be helpful to see the gene constructions illustrated in a figure, perhaps in the supplement.
[Author's response 3-2]
As the referee suggested, we have added a schematic representation showing the construction of chimeric Rad50 in Supplementary Figure S2b.

Other changes in the revised version are as below:
1. To format the manuscript in the journal's style, the abstract has been substantially shortened.
2. Numbers in Supplementary Figure S1b have been re-calculated and modified accordingly.
3. Acknowledgements and Author contributions have been slightly modified.
4. A data availability statement has been included.
5. Typos and grammatical mistakes have been corrected. Sentences in the main and supplementary text have been improved without changing the conclusions.

REVIEWERS' COMMENTS:

Reviewer #1 (Remarks to the Author):

The authors have addressed my concerns and comments carefully and responded in logical and convincing ways. The images are great and the topic of importance. I have no further comments or criticism, and suggest publication of the revised version of the manuscript.

Reviewer #2 (Remarks to the Author):

The revised manuscript has significantly improved. My main objections were properly addressed. However, there are still a few weaknesses outlined below. No experiments are needed to address them.

1. The argument presented in the last paragraph of introduction and second paragraph of discussion is circular. The authors need to at least state another possibility, that the hooks are dynamic and can open both in Rad50 and SMC. All their data show is that the equilibrium is shifted towards the "hook-closed" structures stronger than suggested by the previous Rad50 study.

2. P. 8, l.8: insert "to": were attached to each other…

3. Response to concerns 3 and 4 is insufficient. The authors need to conduct statistical analysis of the data, e.g. using Student's test, and report averages, standard deviations or errors, and p-values to justify their claim that ATP and Nbs1 had an effect of Rad50 conformations. Simply saying that the numbers "slightly increased" (p. 6, l. 4) is insufficient. Please remove the result or analyze it properly.

[Point-by-point responses]

We thank reviewers for their helpful comments and constructive evaluations.
Reviewers' comments (in the black font) and author's responses (in the red font) are as below:
(Page and line numbers refer to locations in the revised manuscript)

Reviewer #1 (Remarks to the Author):
The authors have addressed my concerns and comments carefully and responded in logical and convincing ways. The images are great and the topic of importance. I have no further comments or criticism, and suggest publication of the revised version of the manuscript.

[Author's response 1]
We greatly appreciate his/her positive evaluation on our work.

Reviewer #2 (Remarks to the Author):
The revised manuscript has significantly improved. My main objections were properly addressed. However, there are still a few weaknesses outlined below. No experiments are needed to address them.
1. The argument presented in the last paragraph of introduction and second paragraph of discussion is circular. The authors need to at least state another possibility, that the hooks are dynamic and can open both in Rad50 and SMC. All their data show is that the equilibrium is shifted towards the "hook-closed" structures stronger than suggested by the previous Rad50 study.
[Author's response 2-1]
According to the referee's comment, we have modified the discussion section to clearly state the possibility that both the hook and hinge can open (page 12, line 15-16).

2. P. 8, l.8: insert "to": were attached to each other…
[Author's response 2-2]
We have corrected this typological mistake. In addition, a simple mistake that does not affect any conclusion has been corrected in the figure legend ("$MR_{CC/GG}N$" has been corrected to be "$MR_{CC/GG}$", page 22, line 32).

3. Response to concerns 3 and 4 is insufficient. The authors need to conduct statistical analysis of the data, e.g. using Student's test, and report averages, standard deviations or errors, and p-values to justify their claim that ATP and Nbs1 had an effect of Rad50 conformations. Simply saying that the numbers "slightly increased" (p. 6, l. 4) is insufficient. Please remove the result or analyze it properly.

[Author's response 2-3]

We agree that the statistical analyses are needed to conclude that the presence of ATP or Nbs1 has an effect on the conformation of Rad50. We have removed all these claims from the result section as the referee requested (page 6, first paragraph, line 1 and line 9). We have also removed a speculation that the presence of Mre11 may facilitate the head opening, as it is also needed to be statistically analyzed to conclude (page 7, line 8). The relevant sections have been slightly modified accordingly (page 7, line 8-9).
